# Proposal for a framework for environmental zoning of areas near gold mines based on the distribution of potentially toxic elements, pollution indices, and bioindicators: a case study in Antioquia, Colombia

**DOI:** 10.1007/s10661-024-13079-y

**Published:** 2024-09-14

**Authors:** Julián E. López, Juan F. Marín, Juan F. Saldarriaga

**Affiliations:** 1 Faculty of Architecture and Engineering, Environmental Engineering Program, Institución Universitaria Colegio Mayor de Antioquia, Carrera 78 # 65–46, , 050034 Medellín, Colombia; 2 Department of Civil and Environmental Engineering, Universidad de los Andes, Carrera 1Este, #19A-40, 111711 Bogotá, Colombia; 3 Faculty of Engineering, Universidad de Medellín, 050026, Medellín, Colombia

**Keywords:** Heavy metals, Hazardous waste, Biomonitoring, Environmental risk indicators

## Abstract

Gold mining, even under strict environmental regulations, inevitably causes environmental impacts and liabilities. To address this, the study proposes a framework for environmental zoning around gold mines based on the distribution of potentially toxic elements (PTEs), pollution indices, and bioindicators. Soil samples were collected from municipalities affected by gold mining projects, and concentrations of As, Cd, Pb, and Cr were measured. Then, the Pollution Load Index (PLI) was calculated. A plant model was used for the biomonitoring of PTEs, and the Plant Vigor Index (PVI) was determined. Finally, environmental zoning was proposed through geospatial analysis combining PTEs, PLI, and PVI values. The concentrations of PTEs were as follows: As ranged from 1.7 to 892, Cd from 0.1 to 65.2, Pb from 18.5 to 2345, and Cr from 5.4 to 118.4. Spearman’s rank correlation showed significant relations (ρ > 0.76) between bioindicators and PTE concentrations and PLI. The PVI correlated significantly with PTE concentrations (ρ − 0.41 to − 0.67) and PLI (ρ − 0.65). The municipalities were categorized into three zones: highly contaminated, moderately contaminated, and minimally contaminated. Overall, the environmental zoning maps serve as a management tool for environmental monitoring.

**Supplementary Information:**

The online version contains supplementary material available at 10.1007/s10661-024-13079-y.

## Introduction

Gold mining has significantly contributed to the economic development of many countries. Colombia, ranked as the 5th largest gold producer in Latin America, has notable gold mining operations, with around 40% located in the department of Antioquia (Betancur-Corredor et al., 2018; Bustamante et al., 2016). Despite environmental regulations, gold mining generates well-documented environmental impacts (Betancur-Corredor et al., 2018; Kadivar et al., 2023; Obodai et al., 2024). The environmental impacts are more pronounced in regions, primarily in poor nations, where illicit mining has been on the rise (Chakuya et al., 2023). Mine tailings are the hazardous wastes produced during the extraction of gold ore. The environmental effects of these wastes include significant chemical and dust emissions to the environment (Akoto & Anning, 2021; Orimoloye & Ololade, 2020; Zhu et al., 2022). It has been reported that between 2 and 12 t of mine tailings are produced for every ton of metal extracted from mines (Jiang et al., 2021). The environmental management of mine tailings involves understanding, storing, and ultimately disposing of them. Typically, these waste materials are stored in tailings ponds or dams as a waste management solution. However, the release of potentially toxic elements (PTEs) from these dams can contaminate other environmental components, such as soil, water, and air, thereby affecting the areas surrounding mining sites (Barcelos et al., 2020; Bueno et al., 2009; Orimoloye & Ololade, 2020; Sun et al., 2022). These tailings have the potential to undergo leaching, resulting in the release of PTEs like Cu, Pb, Cd, Ni, Zn, Cr, and As, thereby significantly contributing to environmental degradation (Bueno et al., 2009; Lottermoser, 2010; Wang et al., 2017; Barcelos et al., 2020; Orimoloye & Ololade, 2020; Suppes & Heuss-Aßbichler, 2021). As a result, the accumulation rates of potentially toxic elements exceed those found in natural conditions (background values) (Ngole-Jeme & Fantke, 2017; Teixeira et al., 2019). The harm to the environment that could result from the potential release of metallic pollutants emphasizes the necessity of conducting measurement programs. These initiatives aid in assessing the geographical distribution of pollutants and suggest a method of monitoring them. While campaigns have been conducted in Colombia to measure PTEs, particularly Hg, these have mostly been limited to the Pacific and Caribbean regions (Agudelo-Echavarría et al., 2020; Gutiérrez-Mosquera et al., 2018b; Marrugo-Negrete et al., 2017; Olivero-Verbel et al., 2015; Pinedo-Hernández et al., 2015), and such information is limited for the department of Antioquia, as far as we know. Salazar et al. (2024) have discovered elevated quantities of various elements, including As, Cd, Pb, Cu, Ni, and Cr, in mine tailings from three municipalities in the department of Antioquia.

Different indicators are used to evaluate the specific distribution and monitoring of PTEs in soils and sediments. For example, the total and bioavailable concentrations of these elements, along with their comparison to background values, have been employed (Gallego & Olivero-Verbel, 2021; Marrugo-Negrete et al., 2017). Various pollution indices have been calculated, including geochemical, ecological risk, and human health risk, among others indices (Kowalska et al., 2018; Mosalem et al., 2024; Rodríguez-Seijo et al., 2020; Salazar et al., 2024; Yari et al., 2021). Furthermore, bioindicators have emerged as valuable tools, facilitating the evaluation of biological responses to changes in contaminant levels within the environment. This approach complements conventional indices by providing additional insights into the risk of environmental pollution (Gallego & Olivero-Verbel, 2021; Shi et al., 2017; Yang et al., 2016). The PlantVigor Index (PVI) has been used for biomonitoring of PTEs in soils, e.g., Zhao et al. (2021) found a level of prediction of soil contamination by PTEs of up to 80% using PVI. These authors show decreases in PVI values, from 1000 to values below 10, with increasing concentrations of Ni (above 7.3 mg/kg), Pb (above 20.1 mg/kg), Cd (above 2.7 mg/kg), and Zn (above 12.2 mg/kg) in soils.

The combined use of indicators and biomonitoring is recommended (Salazar et al., 2024). While prior studies on soil contamination using indicator sets and/or bioindicators have primarily focused on assessing contamination levels and toxicological reactions to specific pollutants, few have advocated for environmental zoning of the region (Zaghloul et al., 2020). Implementing zoning measures can help combine diverse indicators to create management tools for improved environmental diagnosis and decision-making. To address this research gap, we propose a framework for investigating soil contamination in areas affected by gold mining. This framework integrates several factors: (i) the concentration of PTEs in soils, (ii) pollution indices, (iii) biomonitoring using a simple vegetation model, and (iv) the identification of special management areas.

Thus, the goals of this study are to (i) reveal the spatial distribution characteristics of As, Cd, Pb, and Cr around gold mining activities, (ii) assess the individual and overall pollution risks of PTEs, (iii) suggest an indicator based on biomonitoring of PTEs in the soil, (iv) propose zoning based on contamination risks and biomonitoring results, and (v) recommend zoning control strategies for potentially toxic element contamination in the three municipalities under study. Unlike other studies, this work proposes an integrative framework for environmental zoning of areas near gold mines based on the distribution of potentially toxic elements, contamination indices, and bioindicators. The results obtained in this study can assist competent authorities in taking actions focused on improving environmental management in the territories impacted by gold mining.

## Materials and methods

### Study areas and sampling

The study was conducted in three municipalities in Colombia: Buriticá (6° 43′ 12″ N, 75° 54′ 27″ O), Puerto Berrío (6° 29′ 26″ N, 74° 24′ 17″ O), and Yalí (6° 40′ 36″ N, 74° 50′ 28″ O) (see Fig. S1 and supplementary description S1).

**Fig. 1:**
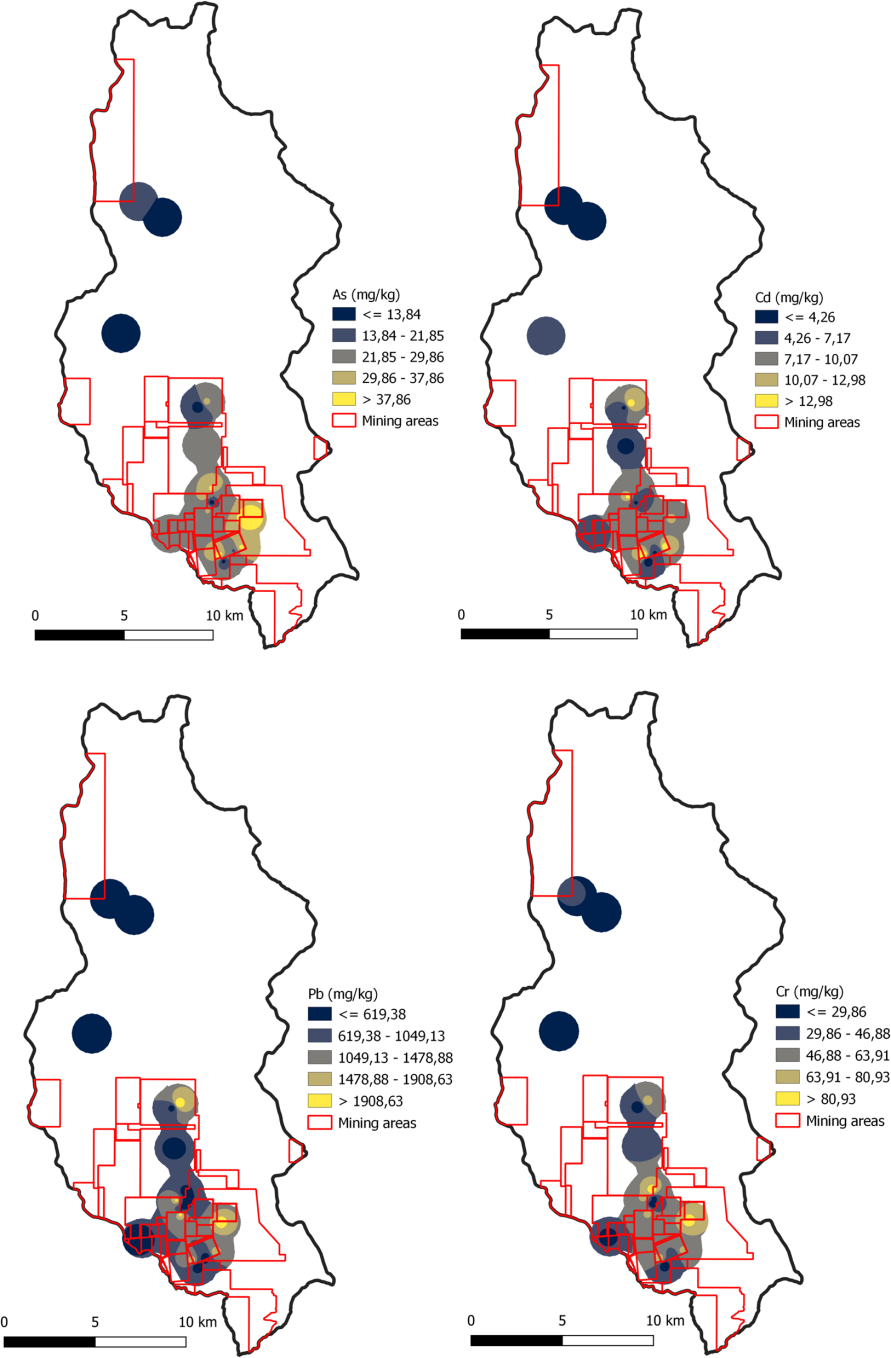
Spatial distribution of PTE concentrations (As, Cd, Pb, and Cr) in soil samples in Buriticá

These municipalities are located in the department of Antioquia, with Buriticá situated in the western sub-region, Puerto Berrio in the middle Magdalena sub-region, and Yalí in the Northeast. Antioquia is renowned for being one of the largest gold producers in Colombia and is also recognized for its significant environmental impacts, particularly on water, soil, and biodiversity, attributed to its extractive practices. Samples were collected in both the direct and indirect impact zones of mining activities. The direct zones included the facilities of mining companies, where gold is often processed, and mining waste is stored in structures known as dams. The indirect zones covered the areas surrounding the mining activities, including population centers and various land uses such as agriculture, industry, and commerce. Soil samples were gathered from the topsoil (0–10 cm) at each location. Each sample, ranging from 500 to 800 g, was carefully placed in Ziploc bags. The samples collected by the municipality were 19 in Buriticá, 23 in Puerto Berrío, and 21 in Yalí (Fig. 1). For data protection reasons, specific coordinates of sampling points and mining company locations are not disclosed in this study.

### Potential toxic element concentration

All samples were air-dried, homogenized, and sieved (2-mm mesh). For the determination of the concentration of potentially toxic elements, 1.0 g of soil sample was subjected to acid digestion using a 1:3 ratio HNO_3_:HCl in an Ethos One Milestone helping Chemist microwave. Then, 10 ml of HNO_3_ and 3 ml of HCl were added. The temperature of the samples was raised to 175 °C in approximately 5.5 min and remained at 175 °C for an additional 4.5 min (U.S. EPA, 2007; Salazar et al., 2024). After digestion, the samples were diluted with ultrapure water and passed through a 0.45-μm membrane filter, and subsequently, the filtrates were used for the determination of elements (As, Cd, Pb, and Cr) by inductively coupled optical emission spectrometry using an Agilent Technologies 5100 ICP-OES equipment (ICP-OES). Quality assurance and quality control protocols were incorporated into the procedure for measuring PTEs (see supplementary description S2).

### Biomonitoring

Radish (*Raphanus sativus* L.) was used as a plant model. Radish is known for being sensitive to elements like Pb (Acosta-Luque et al., 2023) and Cu (B.-Y. Sun et al., 2010). Additionally, this plant has been used for the biomonitoring of complex matrices such as leachates from landfill (Serrano et al., 2023) and compost (López et al., 2023), in which different substances coexist. The radish seeds, obtained from Fercon, had a 90% germination rate and 99% purity. A 1:2 soil-to-deionized water ratio was used to obtain soil–water extracts. At room temperature (25 °C), the samples were shaken for 2 h. The soil samples were centrifuged at 4000 rpm for 15 min. Soil–water extracts were used for phytotoxicity tests. Soil–water extract (6 ml) was used to wet a filter paper (Whatman), which had been previously deposited in a 90-mm Petri dish. Subsequently, 10 seeds were placed in each Petri dish. The Petri dishes were placed in darkness for 24 h, and then, germination was measured. Subsequently, the dishes with radish were stored again in darkness for 24 h, and after that, the radicle elongation was measured. Control seeds were incubated only with deionized water (Acosta-Luque et al., 2023; López et al., 2023; Serrano et al., 2023). Three replicates were performed for each treatment. The incubation conditions of the experimental units were a temperature of 25 °C and darkness. To assess soil phytotoxicity, the germination rate (*G*%) and the radicle elongation (mm) (length) were measured using Eq. 1.

1 $$G\%= \frac{\text{germinated seeds after }24\text{ h}}{\text{total seeds }}\times 100$$

### Data analysis

Statistical analysis was carried out with JMP® Pro version 13.1.0. Prior to analysis, the Shapiro–Wilk test was applied to verify the assumptions of data normality. The PTE data did not fit a normal distribution. To determine statistically significant differences between the analyzed variables for the municipalities of Buriticá, Yalí, and Puerto Berrío, the Kruskal–Wallis tests were used. For non-parametric multiple comparisons, the Steel–Dwass test was used. Spearman’s rank correlation coefficient analysis was used to establish relationships between variables. Cluster analysis was applied to identify different groups, clustering the samples with similar PTE contents, contamination risk values, and biomonitoring values. Ward’s method was used for hierarchical clustering. Principal component analysis (PCA) was applied to determine the key response variables associated with the soil contamination process.

The pollution degree in the soil samples was assessed and compared using the Pollution Load Index (PLI) (Cabrera et al., 1999). This index relies on the Pollution Index (PI), which is determined by dividing the concentration of each PTE in the soil sample (CPTE) (mg/kg) by the background value of that element in the soil (CB) (mg/kg) (Table S1) (Eq. 2). The data used for calculating the PI and PLI were the concentration of each element in each soil sample.

2 $${\text{PI}}_{i}= \frac{{\text{CPTE}}_{i}}{{\text{CB}}_{i}}$$

The values of PI were classified by the following rank of soil pollution: absent (PI < 1), low (1 ≤ PI < 2), moderate (2 ≤ PI < 3), strong (3 ≤ PI < 5), and very strong (PI ≥ 5).

The PLI was obtained as the geometric mean of the measured PIs (Eq. 3).

3 $$\text{PLI}= \sqrt[n]{{\text{PI}}_{1}\times {\text{PI}}_{2}\times {\text{PI}}_{3}\times \dots {\text{PI}}_{n}}$$

Values of PLI < 1 indicate that the potentially toxic element loads are close to the background level, and values above 1 indicate pollution.

The Plant Vigor Index (PVI) was calculated as the product of the *G*% and mean radicle length (Zapata et al., 2024). A higher PVI indicates lower toxic effects, as it reflects greater germination and increased radicle elongation, signifying enhanced plant development (Eq. 4) (Toghueo et al., 2016; Zhao et al., 2021).

4 $$\text{PVI}=\text{mean of radicle length }(\text{cm})\times G\%$$

Spatial analysis was conducted using QGIS 3.24. The spatial dataset used for interpolation comprised 19 points for Buriticá, 23 for Puerto Berrío, and 21 for Yalí. Each point was defined by spatial coordinates and the corresponding concentration of PTEs in the soil, along with estimated values for PLI and PVI. The inverse distance weighting (IDW) method was performed, and the resulting interpolated layers were then clipped using a buffer area associated with each of the sampling points. IDW is a common and convenient interpolation method, which assumes that the value of the unsampled point can be predicted as a weighted average of the known values in the neighborhood. Compared with methods such as kriging interpolation, it is more suitable for data with fewer samples and more uniform distribution (Liu et al., 2023; Lu & Wong, 2008).

## Results and discussion

### Spatial distribution of potentially toxic element concentration

Table 1 shows the minimum, maximum, and median values of the concentration of As, Cd, Pb, and Cr in soil samples from the municipalities of Yalí, Puerto Berrío, and Buriticá. Generally, the lowest PTE concentration values were observed in the municipality of Yalí, as opposed to the municipalities of Buriticá and Puerto Berrío. The concentrations of As, Cd, Pb, and Cr in the municipality of Puerto Berrío were ~ 17-, ~ 12-, ~ 8-, and ~ threefold higher than those in the municipality of Yalí, respectively. On the other hand, the concentrations of As, Cd, and Pb in the municipality of Buriticá were ~ 1-, ~ 4-, and ~ 11-fold higher than those in the municipality of Yalí, respectively. However, for Cr, the concentration in the municipality of Buriticá was ~ onefold lower than that in the municipality of Yalí. When comparing the concentrations of PTEs between the municipalities of Puerto Berrío and Buriticá, the concentrations of As, Cd, and Cr in Puerto Berrío were ~ 32-, ~ 4-, and ~ fourfold higher than in Buriticá, respectively. Conversely, the concentration of Pb was ~ 1.3-fold higher in Buriticá compared to Puerto Berrío. The concentrations of PTEs found in this study were ~ 8-, ~ 34-, and ~ 4.4-fold higher than the values recorded for Cd, Pb, and Cr, respectively, in soils near gold mines in other countries (e.g., Bolivia, Nigeria, South Africa, and China) (Hou et al., 2023). In soils affected by gold mining in Ecuador (Romero-Crespo et al., 2023), the concentrations of As (23.54–421.74 mg/kg) and Cr (77.24–101.04 mg/kg) showed similar ranges to those found in this study. However, for elements like Cd and Pb, this study reports concentrations ~ 3.6- and ~ 16-fold higher than those of these same authors. In comparison to other studies conducted in Colombia, Pb and Cd values in this study were ~ 2.7- and ~ 2.3-fold higher than those found for soils near a gold mine in northern Colombia (Durante-Yánez et al., 2022). In contrast, the Pb and Cd values found in this study were ~ 4975- and ~ 188-fold higher than those found in agricultural soils downstream of a gold mining district in northern Colombia (Marrugo-Negrete et al., 2017).

**Table 1:** Statistical summary of potentially toxic elements (PTEs) (As, Cd, Pb, and Cr) concentration (mg/kg) in soil samples from Yalí, Puerto Berrío, and Buriticá

Soil samples		As	Cd	Pb	Cr
Yalí (*n* = 21)	Minimum	8.1	0.1	18.5	5.4
	Maximum	35.5	4.6	201.3	118.4
	Median	11.3	1.3	89.0	19.0
Puerto Berrío (*n* = 23)	Minimum	1.7	0.1	13.0	65.4
	Maximum	892	65.2	1908	301
	Median	98.4	9.7	89.7	109
Buriticá (*n* = 19)	Minimum	5.8	0.5	189	5.4
	Maximum	49.8	18.2	2345	102.3
	Median	22.9	5.4	422	34.8
Soil clean-up standards (SCSs) (Provoost et al., 2006)					
SCSs for residential land use (mg/kg)		34	15	10,175	419
SCSs for industrial land use (mg/kg)		173	254	14,298	1150

**Fig. 2:**
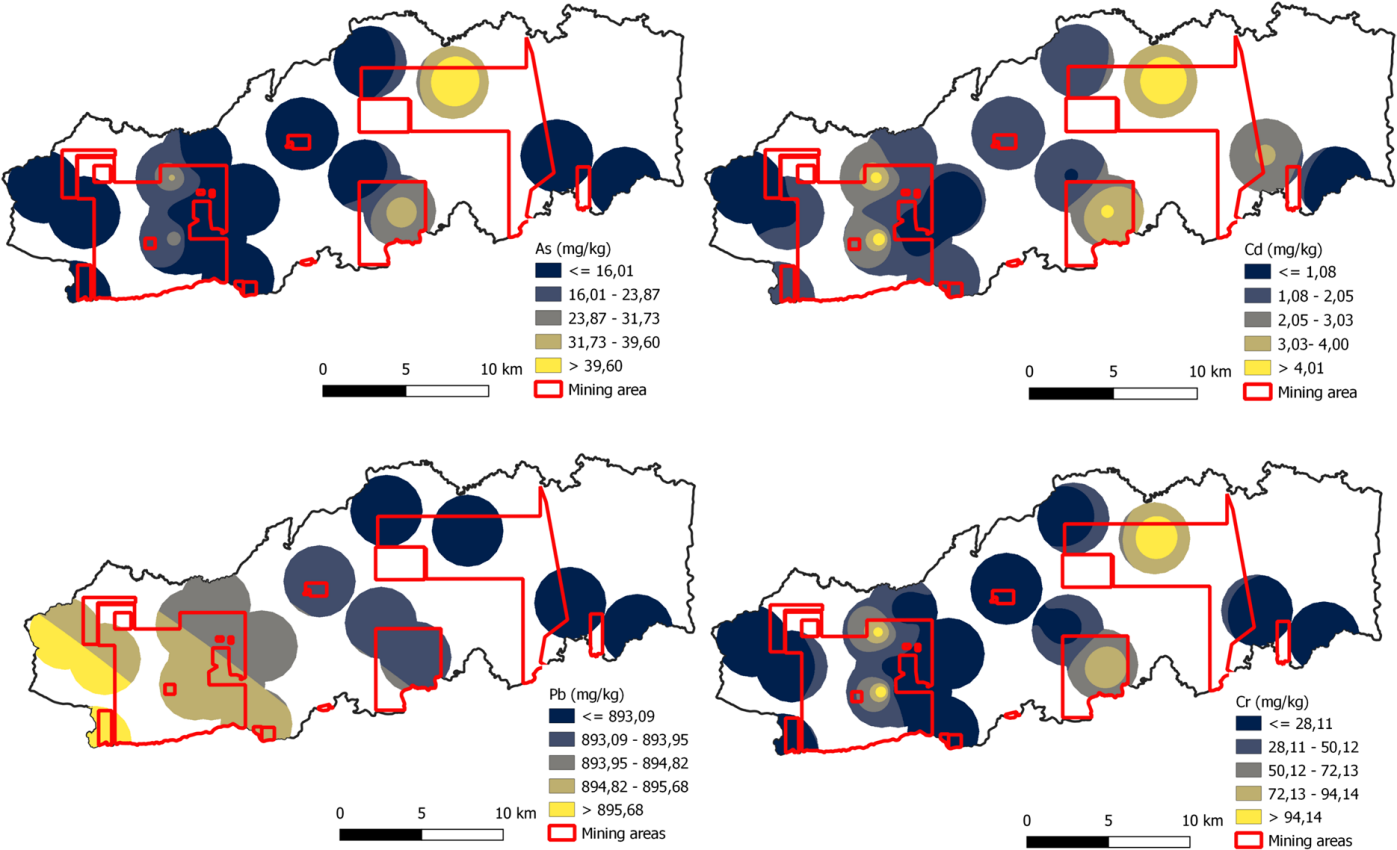
Spatial distribution of PTE concentrations (As, Cd, Pb, and Cr) in soil samples in Yalí

**Fig. 3:**
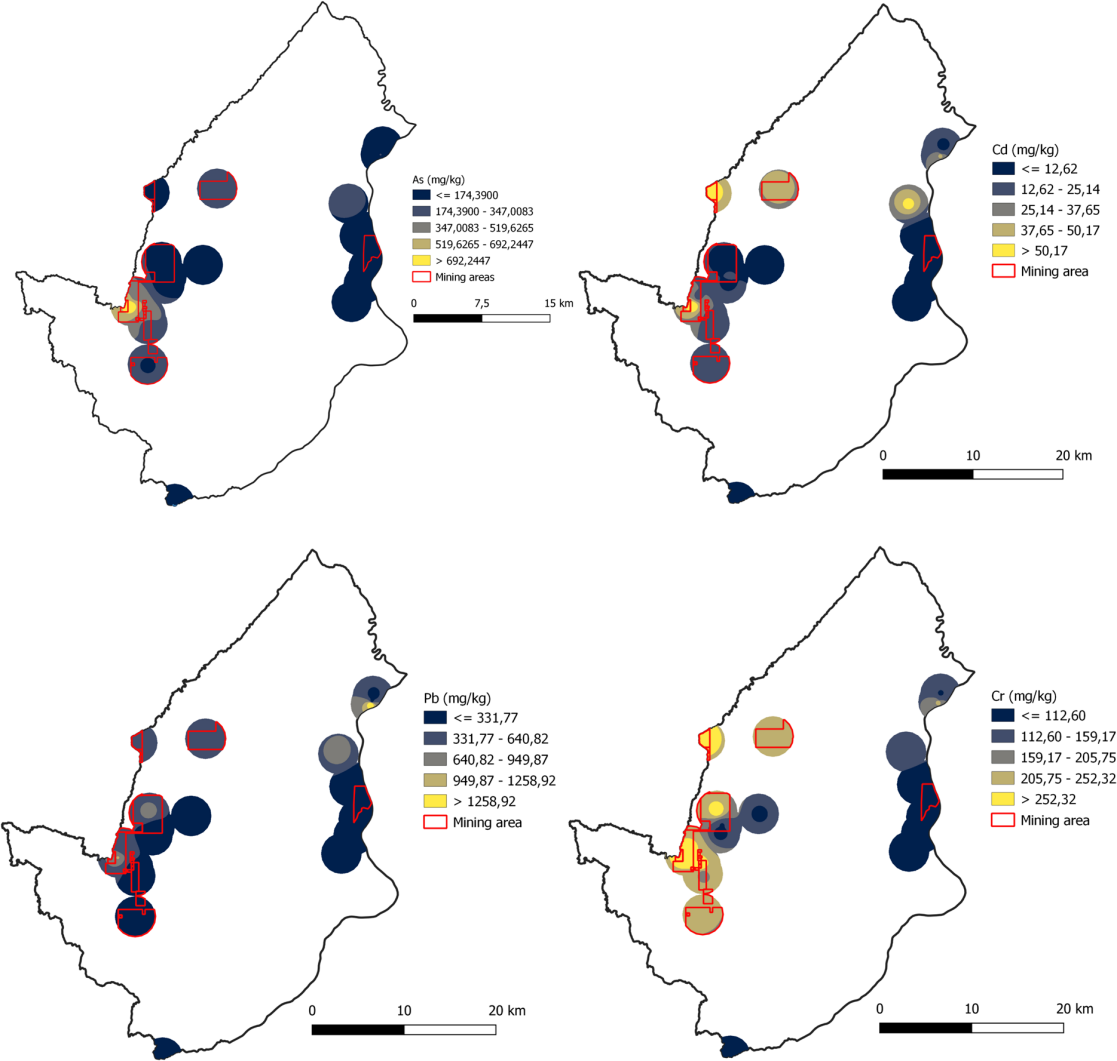
Spatial distribution of PTE concentrations (As, Cd, Pb, and Cr) in soil samples in Puerto Berrío

Figures 1, 2, and 3 show the spatial distribution of PTEs in Buriticá, Yalí, and Puerto Berrío, respectively, obtained using the IDW interpolation method. The maps reveal multiple hot spots linked to heightened concentrations of PTEs in specific regions. In Yalí municipality, the concentration values of As, Cd, Pb, and Cr in hot spots were ~ 2.3-, ~ 4.4-, ~ 2.8-, and ~ 3.3-fold higher compared to the areas with lower values or cold spots, respectively. For Puerto Berrío municipality, the concentration of As, Cd, Pb, and Cr in the hot spots was ~ 3.9-, ~ 3.4-, ~ 3.8-, and ~ 2.2-fold higher compared to the cold spots, respectively. In Buriticá municipality, the values of As, Cd, Pb, and Cr in the hot spots were ~ 2.9-, ~ 3.0-, ~ 3.1-, and ~ 2.6-fold higher compared to the cold spots, respectively. Overall, the maps indicate that hot spots are primarily associated with gold mining areas, particularly those with active mining titles, suggesting an increase in PTE concentrations near the gold mines. When comparing municipalities, the largest hot spots for As, Cd, and Cr were located in Puerto Berrío municipality. The concentrations of As, Cd, and Cr were ~ 23.0- and ~ 16.8-fold higher, ~ 12.5- and ~ 4.2-fold higher, and ~ 2.7- and ~ 3.4-fold higher in Puerto Berrío compared to Yalí and Buriticá municipalities, respectively. On the other hand, the largest hot spot for Pb was found in Buriticá municipality. The Pb concentration in Buriticá municipality was ~ 10.9-fold higher compared to Yalí municipality and ~ 1.7-fold higher compared to Puerto Berrío municipality. Due to the number of sampling points and the distance between them, the IDW analysis or any other interpolation method may have limitations in predicting the response surface. It is important to subsequently conduct new monitoring campaigns to determine the distribution of PTEs over a larger area.

Several factors explain the distribution of PTE concentrations. On one hand, the weathering of rocks contains abundant minerals such as galena (PbS), tennantite (Cu_12_As_4_S_13_), proustite (Ag_3_AsS_3_), sphalerite (ZnS) (Cd-enriched variant), chromite (FeCr_2_O_4_), and crocoite (PbCrO_4_). The geochemical map of Colombia shows the abundance of elements such as As, Cd, Pb, and Cr in the lithology (soils, sediments, rocks) in the three municipalities studied (Fig. S2). The direct influence zones of the mining projects are linked to the hotspots identified on the map, most likely due to activities related to gold extraction and processing. These zones include inert waste deposits, tailings dams, gold beneficiation plants, and the exploitation of the deposit. Previous reports for Yalí, Buriticá, and Puerto Berrío have shown concentrations of Pb (37.0–9726 mg/kg), Cd (3.4–65.2 mg/kg), As (27.0–3555 mg/kg), and Cr (13.0–281 mg/kg) in gold mining tailings (Salazar et al., 2024). When comparing these values with those reported for other regions, it is observed that, for example, the concentrations of Pb and As exceed the values reported for tailings from gold and copper mining in Chile, which are 15–272 mg Pb/kg and 10–119 mg As/kg (Medina Tripodi et al., 2019), respectively. On the contrary, for elements like Cd and Pb, the values reported for Yalí, Buriticá, and Puerto Berrío are below the values reported for gold mines in Brazil, which are 143 mg Cd/kg and 23,337 mg Pb/kg, respectively (Barcelos et al., 2020). It is important to highlight that the concentrations of PTEs present in mining tailings will depend mainly on the geochemistry of the area. The PTEs present in tailings can be mobilized to other areas due to acid mine drainage, direct surface runoff, and dust emissions, degrading adjacent soils, contaminating watercourses, and potentially affecting living organisms (Bueno et al., 2009; Hou et al., 2023; Orimoloye & Ololade, 2020; Sun et al., 2022). The mobilization of PTEs from mining tailings to the indirect influence areas of mining projects will depend on the particle size and wind. For instance, it has been reported that higher concentrations of PTEs are found in smaller particle sizes (e.g., < 63 μm) (airborne material that is generated from the tailings), which are easily transported by the wind (Bailey et al., 2021). On the other hand, topographic factors such as the slope favor direct surface runoff, increasing the mobilization of material deposited in the soils (Wu et al., 2023). In this case, the municipalities of Buriticá, Yalí, and Puerto Berrío are located in the Andean region of Colombia, which is characterized by a mountainous terrain and steep slopes (Fig. S3).

The indirect influence areas of mining projects include other land uses (agricultural, residential, and industrial), which are permitted both within the mining title concession area and within its perimeters. In agricultural areas, crops such as corn, beans, tomatoes, and lettuce were observed. Residential land use areas include municipal centers, small population settlements, and independent houses in rural areas. Industrial land use areas feature activities such as commerce and metal-mechanical factories. It is possible that other activities in these areas have contributed to the increase in PTE concentrations. For example, it has been observed that activities such as the transportation of heavy machinery, industrial activities, and commercial activities could raise the levels of Pb and Cd in soils (Castañeda-Restrepo et al., 2022; Trujillo-González et al., 2019). In the three municipalities, metalworking activities were observed, as well as the traffic of heavy machinery, which could have had an influence on the concentrations of PTEs (Castañeda-Restrepo et al., 2022). Likewise, agriculture has been identified as an important factor favoring the concentration of metals such as Cd, As, and Cr, among others, in soils (Adhikari et al., 2021; Atafar et al., 2010). Several anthropogenic activities, beyond gold mining, could explain the increase in the concentration of elements in soils. Further analyses are needed to understand the factors influencing the spatial variation in the concentration of these elements associated with the diversity of land uses in the territories.

To better understand the potential implications of the increase in As, Cd, Pb, and Cr in the studied areas, the concentration values of the PTEs found in this study were compared with soil clean-up standards (SCSs) (mg/kg) for residential and industrial use (Provoost et al., 2006) (Table 1). The authors compiled SCSs from various countries: Belgium, the Netherlands, Germany, France, Switzerland, Norway, Great Britain, Canada, Sweden, and the U.S.A. Finally, the author proposed SCS values based on the statistical processing of the reference values. International values were used since Colombia lacks specific regulations for limiting the values of PTEs in soils. In Buriticá, 26% of the samples exceeded the recommended limit for As in soil intended for residential use. Regarding Cd, 11% of the samples reported concentrations above the recommended value for residential areas. Additionally, 53 and 32% of the samples surpassed the concentration for Pb in residential and industrial zones, respectively. In Yalí, the concentration of As for residential use exceeded the recommended limit in 5% of the samples. For Cd and Pb, none of the analyzed samples exceeded the reference values in residential or industrial zones. For Puerto Berrío, 65 and 30% of the samples had concentrations of As above the standard for residential and industrial zones, respectively. A total of 43% of the samples exceeded the recommended values for Cd in residential areas. Additionally, 17 and 4% of the samples surpassed the recommended limits for Pb in residential and industrial soils, respectively. The concentrations of Cr did not exceed the recommended limits in any of the municipalities. Overall, Puerto Berrío exhibited the highest percentage of samples exceeding the recommended concentrations of PTEs in soils. The PTE values above soil clean-up standards can be associated with enrichment due to mining activity and other land uses, as discussed previously. Especially in sampling areas near gold mining sites, samples show values that exceed these standards. In contrast, soil samples taken in areas farther from mining projects show lower levels of enrichment. For example, in the northern region of Colombia, the enrichment of PTEs associated with open-pit gold and ferronickel mining, as well as the use of agricultural inputs, has been demonstrated (Marrugo-Negrete et al., 2017).

### Pollution risk

**Fig. 4:**
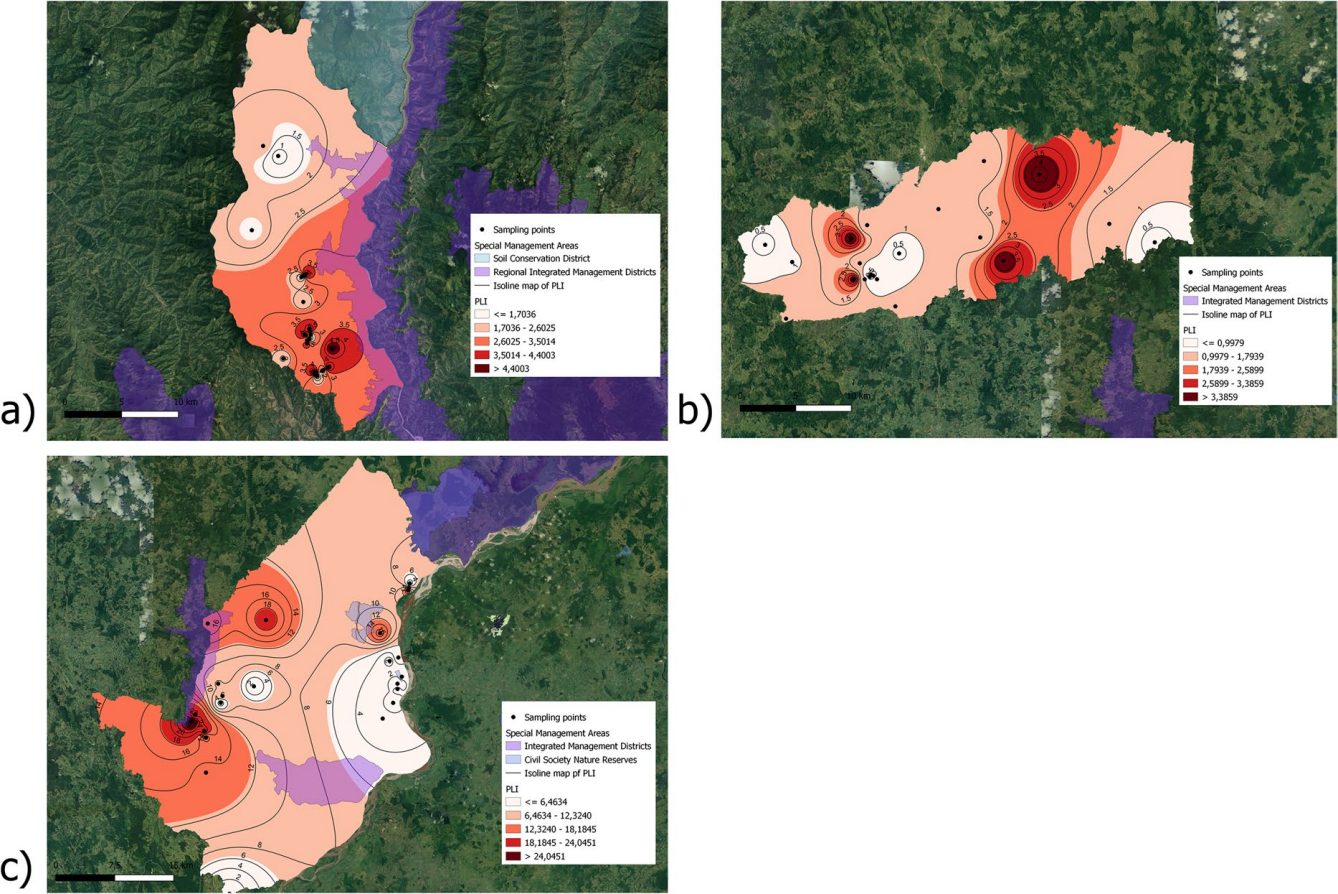
Pollution risk maps for Buriticá (**a**), Yalí (**b**), and Puerto Berrío (**c**) based on the Pollution Load Index (PLI)

The corresponding PI values and maps illustrating pollutant risk zoning are presented in Table S2 and Fig. 4, respectively. The Kruskal–Wallis test showed statistically significant differences in PI values for As among the three municipalities. For Cd, significant differences were found in PI values between the municipalities of Yali and Puerto Berrio and Yalí and Buriticá, but not between Puerto Berrío and Buriticá. Regarding PI for Pb and Cr, differences were found between the municipalities of Puerto Berrío and Buriticá and between Yalí and Puerto Berrío, but not between Yalí and Buriticá. For Buriticá, the calculated PI values ranged from 1.0 to 8.3, 1.4 to 52.0, 0.2 to 2.9, and 0.1 to 1.5 for As, Cd, Pb, and Cr, respectively. Specifically, regarding As, the PI was classified as very strong in approximately 37% of the samples, while the remaining samples exhibited variations between low, moderate, and strong risk. Specifically for Cd, 95% of the samples were categorized as very strong, whereas for Pb and Cr, the majority of the samples fell into the absent, low, and moderate risk categories. For Yalí, the observed PI ranges were 1.4 to 5.9, 0.1 to 13.2, 0.2 to 3.4, and 0.1 to 1.7 for As, Cd, Pb, and Cr, respectively. According to the risk classification, PI values for As spanned from low, moderate, strong, and very strong. In the case of Cd, Pb, and Cr, there were PI values classified as absent, as well as others marked as moderate, strong, and very strong. In Puerto Berrío, the PI values varied from 0.3 to 148, 0.0 to 186, 1.9 to 8.6, and 0.9 to 4.3 for As, Cd, Pb, and Cr, respectively. In this municipality, about 61% of the soil samples indicated PI values classified as very strong for As and Cd. For Pb and Cr, the PI values were categorized as moderate, strong, and very strong. In general, at most of the sampled points, the PI values indicated a significant level of risk. This suggests an enrichment of As, Cd, Pb, and Cr, as the concentrations exceeded the background values of elements (Cabrera et al., 1999). Particularly, As and Cd were the elements presenting the greatest risk of contamination. These points were predominantly situated in areas directly influenced by mining activities.

Pollution zoning maps based on PLI indicate that in Buriticá, 16% of the samples showed a low risk of contamination from the analyzed PTEs, while the remaining 84% of samples indicated a risk of contamination. In Yalí, 38% of the samples reported a risk of environmental contamination, and the remaining 62% did not indicate a risk of contamination. And in Puerto Berrío, 87% of the samples indicated a risk of contamination, and only 13% indicated a low risk of contamination. According to these results, Buriticá and Puerto Berrío exhibit a higher level of contamination associated with the enrichment of As, Cd, Pb, and Cr in the soils compared to Yalí, and As and Cd contribute the highest level of contamination to the PLI for the three municipalities. The PLI reveals that in Buriticá, 16% of the soil samples exhibit a low risk of contamination, while the remaining 84% of soil samples indicate a risk of contamination. In Yalí, 38% of the samples report a risk of environmental contamination, with the remaining 62% not indicating contamination risk. Conversely, in Puerto Berrío, 87% of the samples indicate a risk of contamination, while only 13% indicate a low risk of contamination.

Locations requiring special management inside or close to the three municipalities under study were noted in Fig. 4. All these sites under special administration are regarded as part of the Colombian National Natural Park System, which is endowed with legislative protection and special management requirements from an environmental standpoint. One of the identified areas is the Regional Integrated Management Districts, which serve as a model of rational use. This model facilitates activities aimed at guaranteeing the economic, social, and cultural well-being of human beings through the sustainable use of resources. Another area identified is the Soil Conservation Districts, representing geographic zones where strategic ecosystems at a regional scale maintain their function, although their structure and composition have been modified. These districts contribute significantly to the generation of environmental goods and services. Additionally, Civil Society Natural Reserves was identified as private areas that preserve a sample of a natural ecosystem. These reserves are managed under the principles of sustainability in the use of natural resources and are intended for sustainable use.

It is observed that in Buriticá, a part of the area corresponding to the integrated management district overlaps with one of the areas with the highest PLI values (3.5 to 4.4). Likewise, there is proximity between an area with PLI values between 1.7 and 2.6 and the area corresponding to the soil conservation district. For Puerto Berrío, areas with PLI values between 6.4 and greater than 24 are observed in the areas where the Integrated Management Districts and Natural Reserves of Civil Society are located. These findings underscore the need for environmental authorities to improve monitoring of the mobility of potentially toxic metals in the territory. The increasing levels of pollution in these areas represent a threat to the provision of ecosystem services and the conservation of the biodiversity that resides in them.

### Biomonitoring

The results for *G*% and radicle length for each soil sample are presented in Table S3. Indices such as PLI and ecosystem risk indices like NIPI, RAC, and GAI serve as indicators of contamination levels in soils and/or sediments. However, they do not represent the biological systems’ response to variations in contaminants concentration (Kowalska et al., 2018; Mosalem et al., 2024; Rodríguez-Seijo et al., 2020; Salazar et al., 2024; Yari et al., 2021). The use of plants and/or other organisms has been employed to complement mathematical indices (Nannoni & Protano, 2016; Rodríguez-Seijo et al., 2020; Salazar et al., 2024). In this study, biomonitoring was proposed. Previous research has shown that radishes have exhibited significant responses to changes in contamination levels associated with potentially toxic elements and other pollutants in complex matrices (Acosta-Luque et al., 2023; Bhat et al., 2011; López et al., 2023; Serrano et al., 2023; Tiwari et al., 2013).

The Kruskal–Wallis test showed statistically significant differences in *G*% values and root elongation between the municipalities of Yalí and Buriticá and Yalí and Puerto Berrío, but not between Buriticá and Puerto Berrío.

The *G*% and radicle length were significantly correlated with the concentration of PTEs and PLI values (Table S4). Biometric parameters and changes in seed germination are reactions to the stress induced by metal exposure (Gill & Tuteja, 2010; Tiwari et al., 2013; Salazar et al., 2024). Therefore, the observed changes in the length and *G*% can be linked to exposure to As, Cd, Pb, and Cr present in the soil samples. Similar results were found by Salazar et al. (2024) about the concentrations of PTEs in mine tailings samples. Using *Phaselou vulgaris* L., changes in biochemical biomarkers correlated significantly with biometric variables measured in *Phaselou vulgaris* L. plants and with the concentration of As, Cd, Cr, Pb, Ni, and Cu (Salazar et al., 2024). Likewise, Lopez et al. (2024) found a significant correlation between changes in biochemical biomarkers and EPTs concentration for radish seedlings directly planted in contaminated soils. The PVI has been used as a bioindicator to assess the phytotoxicity of heavy metals on seedling growth (Zhao et al., 2021). PVI correlated significantly with both *G*% and elongation, as well as with the concentration of PTEs. These results suggest that PVI is an indicator of variation in the concentrations of PTEs in the soil. A decrease in PVI indicates higher toxicity, while an increase in PVI indicates lower toxicity.

**Fig. 5:**
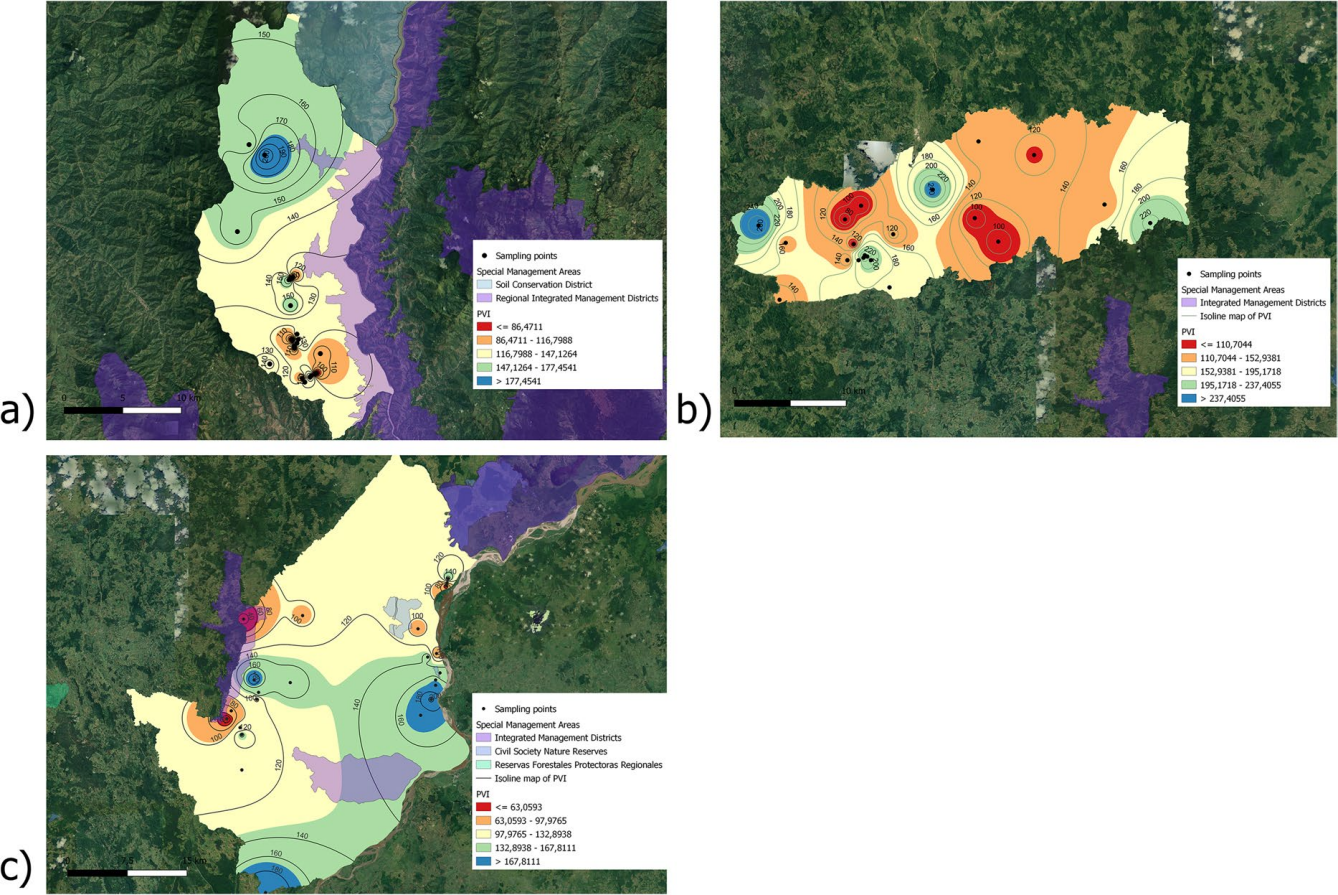
Zoning maps for Buriticá (**a**), Yalí (**b**), and Puerto Berrío (**c**) based on Plant Vigor Index (PVI)

PVI was utilized for constructing toxicity risk zoning maps in the three municipalities (Fig. 5). Values below 80 can be considered as areas where there is a significant impact on the germination and development of radish seedlings (phytotoxicity) (López et al., 2023). Areas with lower PVI values coincide with the hot spots of contamination delimited using the PLI and with the distribution of PTEs. These findings suggest, together with the correlation analysis, that there is a significant relationship between the biological model used and the changes in the concentration of PTEs in the soil samples. Thus, it is suggested that the PVI be used for biomonitoring of PTEs in the three municipalities. Zhao et al. (2021) used biometric parameters and PVI calculation to biomonitor the concentration of EPTs in soils. Similar to the results of this study, *G*% and radicle elongation responded significantly to changes in the concentration of EPTs in soils. In another study, Calvelo Pereira et al. (2010) found that *G*% was not a good bioindicator for soils contaminated with hexachlorocyclohexane. On the contrary, these authors found that the germination rate, biometric parameters, and PVI were better bioindicators for diagnosing contaminated soils. Also, for Pb-contaminated soils, Acosta-Luque et al. (2023) found that the *G*% of radish seeds was a good indicator to determine the toxicity of the soil after the implementation of the treatment, suggesting this bioindicator for ex-post evaluation. For other environmental matrices, landfill leachate, Serrano et al. (2023) found that the *G*% and radicle elongation of radish responded significantly to changes in the concentration of EPTs (e.g., Cd, Pb, Zn, Cr) in the leachate before and after treatment. Results of this study, support that the use of bioindicators such as *G*% and other biometric parameters are useful for environmental biomonitoring.

Especially in Puerto Berrío, the PVI hot spots coincide with parts of the areas designated as Regional Special Management Districts. This supports the notion that these special management zones are vulnerable to pollution processes that could jeopardize ecosystems. It is important to consider the limitations associated with the biomarkers determined in the study, as their assessment was not conducted in situ. Therefore, ex situ environmental conditions may lead to changes in the response of the plant model. Additionally, it is suggested to use these biomarkers employing native plants from the area and conduct in situ evaluations.

**Fig. 6:**
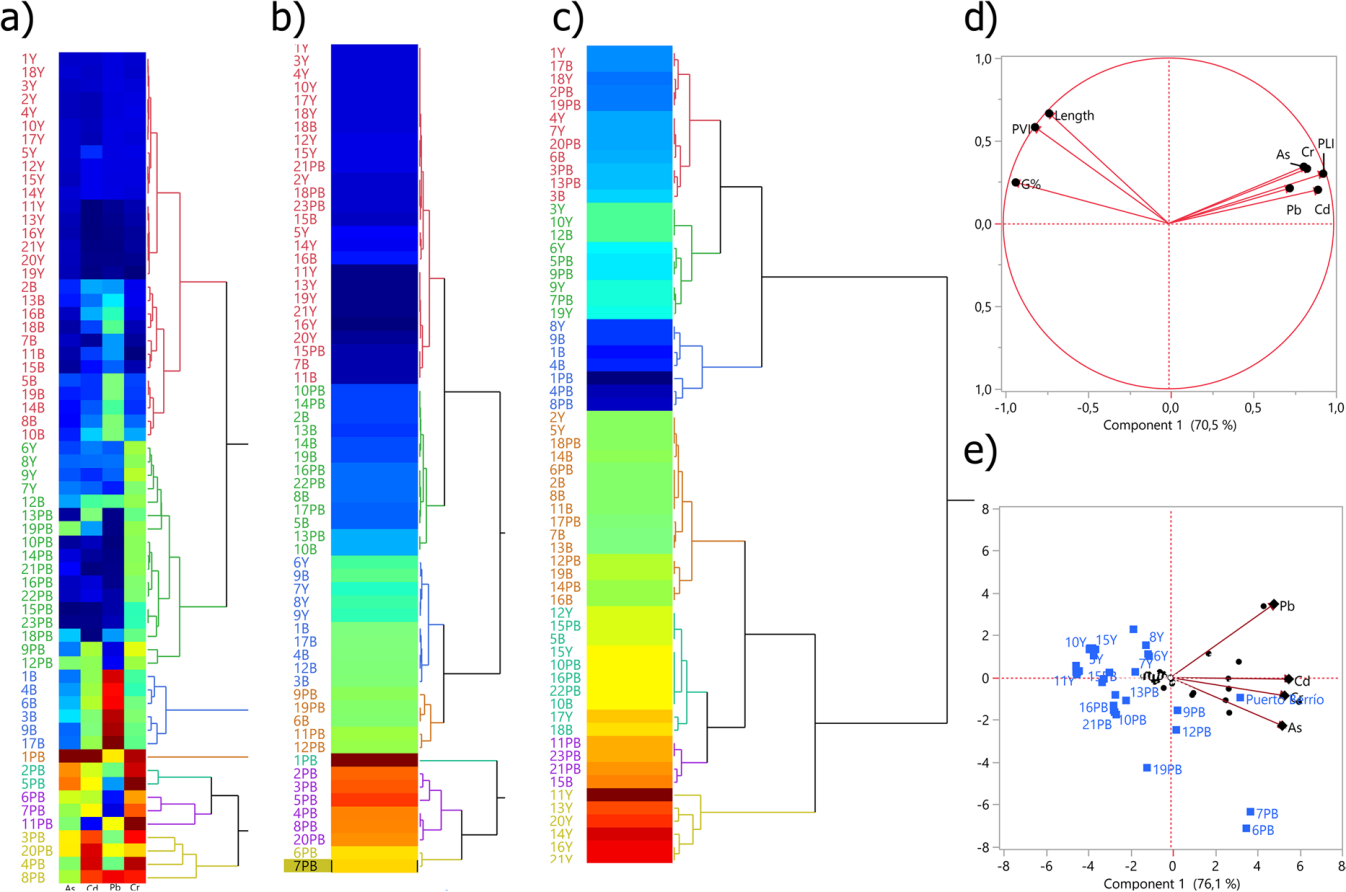
Cluster and principal component analysis (PCA). Dendogram obtained by hierarchical clustering analysis for **a** potential toxic elements, **b** Pollution Load Index (PLI), and **c** Plant Vigor Index (PVI). The heat maps represent the numerical variation from lower (blue) to higher (red) values. The variation in the color of the letters and lines of the dendrograms represents the clusters that were formed. PCA results. **d** Loading plot of the two components. **e** Biplot of sampling sites and concentration of PTEs. The numbers and letters represent the codes of each soil sample. The letter Y represents Yalí soil samples. The letter B represents Buriticá soil samples. The letters PB represent Puerto Berrío soil samples

Cluster analysis results indicate the formation of clusters based on the concentration of PTEs (Fig. 6a and Table S5), PLI (Fig. 6b and Table S6), and PVI (Fig. 6c and Table S7). Most of the samples from Puerto Berrío and Buriticá were grouped in the clusters with the highest concentration of PTEs and the highest PLI values. On the contrary, most of the samples from Yalí were located in clusters with lower values of PTEs and lower PLI values. The cluster formed from the highest PVI values was formed by samples from Yalí. As PVI values decreased, the clusters began to be formed by samples from Puerto Berrío and Buriticá and a smaller number of samples from Yalí. As in the correlation analysis, the samples grouped in clusters that indicated a higher risk of contamination were grouped in clusters that grouped samples with reductions in PVI values.

To further assess the extent of PTE contamination in the study areas and identify the source, PCA was used (Fig. 6d and e). This technique clusters variables into groups, such that variables belonging to one group are highly correlated with one another. The PCA results lead to a reduction of the initial dimension of the dataset to two components explaining 86.1% of the data variation. As, Cd, Pb, Cr, and PLI were involved in the first component, whereas the second component included *G*%, length, and PVI. The grouping of variables in the first component suggests a general source of the PTEs. As discussed earlier, this source could be the dispersion of contamination from accumulated mine tailings in the area. The opposite location of the variables involved in biomonitoring suggests that there is a response that decreases with the increase in PTE concentration and PLI. To facilitate environmental zoning, the PCA analysis suggests that zones with higher PLI values and lower *G*% and PVI values are of higher priority, due to higher contamination and toxic response of the plant model. On the other hand, zones with lower PLI values and higher *G*% and PVI values indicate a lower level of pollution and a lower phytotoxic response.

### Environmental zoning

**Fig. 7:**
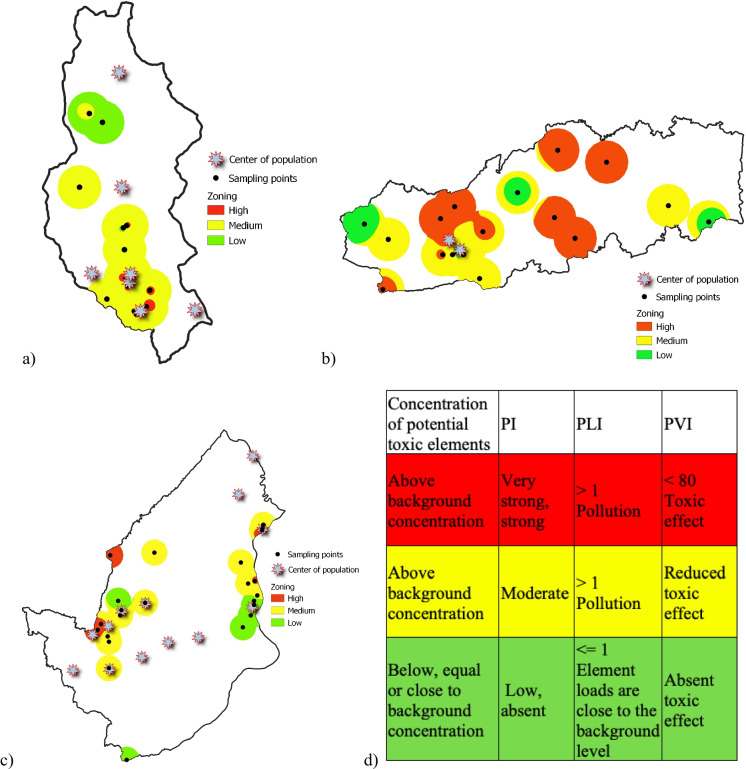
Environmental zoning maps for Buriticá (**a**), Yalí (**b**), and Puerto Berrío (**c**) and proposed environmental zoning framework (**d**)

The simultaneous application of a set of different methods for assessing soil contamination, together with zoning methods, provides a better understanding of the extent of contamination and the associated risks. In this study, for the environmental zoning, each of the municipalities was divided into three regions based on the concentration of PTEs and the PI and PLI values, as well as the PVI value. The resulting areas were labeled as red area, yellow area, and green area (Fig. 7).

The red area refers to a zone with high concentrations of PTEs, where both PI and PLI values indicate enrichment of these elements in the soil and severe contamination. Additionally, PVI values indicate toxic effects on the studied plant model. In the case of Buriticá and Puerto Berrío, the red area also includes zones where special management areas overlap with contamination risk zoning and biomonitoring. This region requires a human intervention strategy focused on remediation to mitigate the risk to the ecosystem and the population from potential exposure to PTEs. Previous studies in the Pacific and the Colombian Caribbean have identified areas highly degraded by gold mining that necessitate human intervention for recovery. For instance, Gutiérrez-Mosquera et al., (2018a, 2018b) highlighted metals like Pb as key elements that need management to diminish the risk to ecosystems. Similarly, Marrugo-Negrete et al. (2017) observed that downstream of a zone highly impacted by gold mining and ferronickel mining and the presence of metals such as Hg and Ni led to environmental impacts on agricultural soils. It is essential to implement decontamination processes using biological and/or physicochemical alternatives in these areas. Phytoremediation has been proposed as an alternative for remediating soils impacted by gold mining in Colombia (Durante-Yánez et al., 2022; Marrugo-Madrid et al., 2021). The yellow area refers to a zone where, although there may be enrichment by PTEs, the PI values indicate moderate levels of contamination. This area needs to be protected and restored naturally. The green area refers to the region that is currently low in pollution (PI and PVI values) and needs to focus on prevention. This region consists of areas with low potential pollution risk and low potentially toxic elements concentrations, below the background value to very close to it.

## Conclusions

This study presents, for the first time, the concentration and spatial distribution of As, Cd, Pb, and Cr in three municipalities: Buriticá, Yalí, and Puerto Berrío, located in the Andean region of Colombia. A methodology for PTE biomonitoring based on phytotoxicity biochemical biomarkers using a rapid-response plant model is proposed. PTE concentrations exceeded background levels in the soil, particularly in regions near gold mining areas. The environmental zoning maps serve as a management tool for environmental monitoring in the area. The study area was divided into three zones combining PTE concentrations with PI, PLI, and PVI values. Prevention, control, and mitigation strategies are proposed according to the environmental zoning of the territory to provide a scientific reference for managing some environmental impacts generated by gold extraction activities. With the results obtained, it would be pertinent to analyze in a future study the impact on the flora and fauna of the area, and in order to determine the impact, this could have on the territory and its socio-economic activities. Knowledge about the presence of potentially toxic elements (PTEs) in areas surrounding gold mining projects provides important information regarding potential risks they may pose. However, further studies are necessary to determine the potential risk to human health.

## Supplementary Information

Below is the link to the electronic supplementary material.

Supplementary file1 (DOCX 6.62 MB)

## Data Availability

No datasets were generated or analysed during the current study.
