# Supplementary material for: Proposal for a framework for environmental zoning of areas near gold mines based on the distribution of potentially toxic elements, pollution indices, and bioindicators: a case study in Antioquia, Colombia
Source: Environ Monit Assess. 2024 Sep 14;196(10):933. doi: 10.1007/s10661-024-13079-y (PMC11399291; doi:10.1007/s10661-024-13079-y)
Supplement: Supplementary file 1 — Supplementary file1 (DOCX 6.62 MB) [file 10661_2024_13079_MOESM1_ESM.docx]

**Supplementary information for:**

**Proposal for a framework for environmental zoning of areas near gold mines based on the distribution of potentially toxic elements, pollution indices, and bioindicators. A case study in Antioquia, Colombia**

Julián E. López ^a*^, Juan F. Marín ^a^, Juan F. Saldarriaga ^b^

^a^ Faculty of Architecture and Engineering, Environmental Engineering Program, Colegio Mayor de Antioquia, Carrera 78 # 65 – 46, 050034, Medellín, Colombia.

^b^ Department of Civil and Environmental Engineering, Universidad de los Andes, Carrera 1Este #19A-40, 111711, Bogotá, Colombia.

***Corresponding author:**

Julián E. López

E-mail: [julian.lopez@colmayor.edu.co](mailto:julian.lopez@colmayor.edu.co)


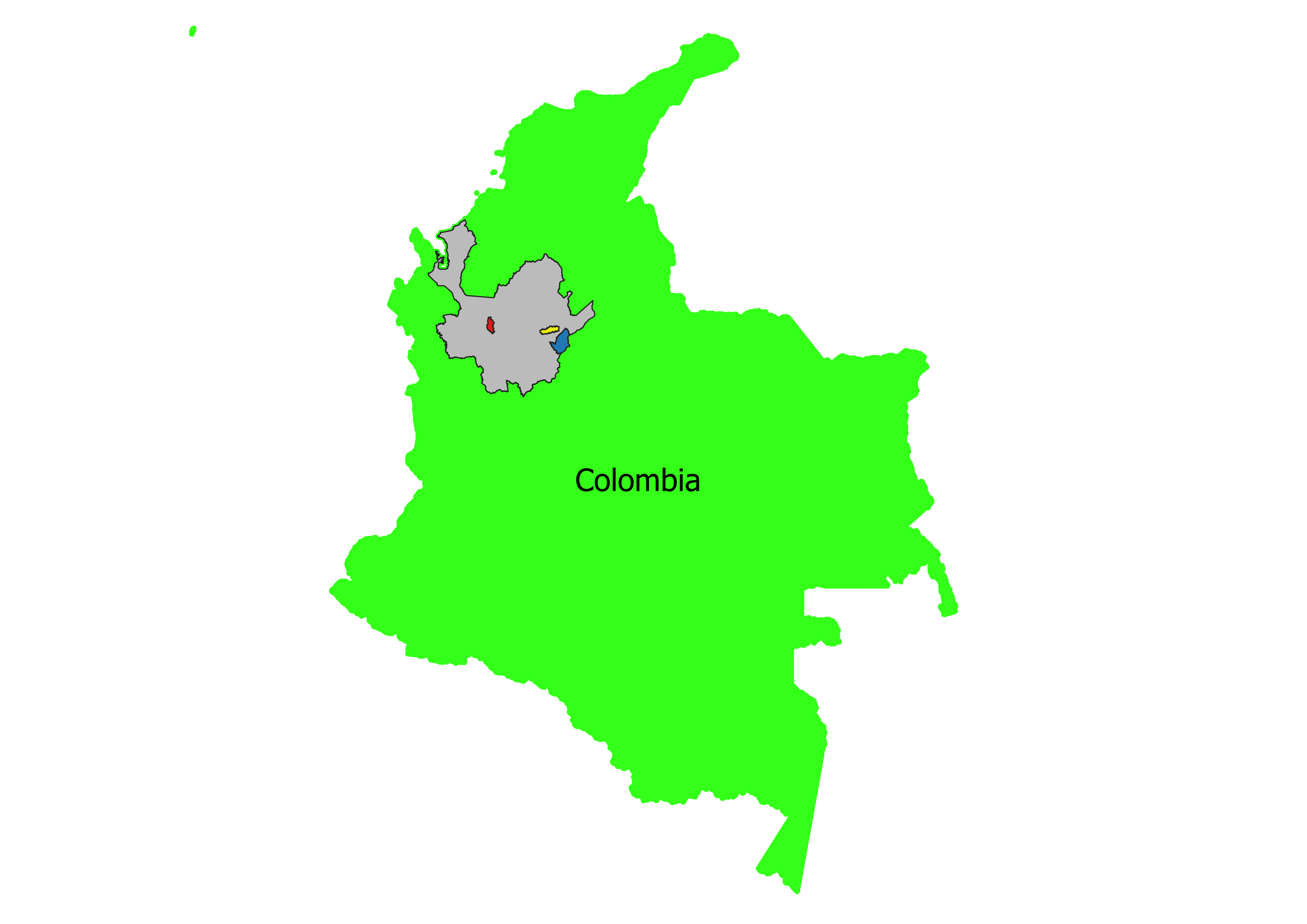

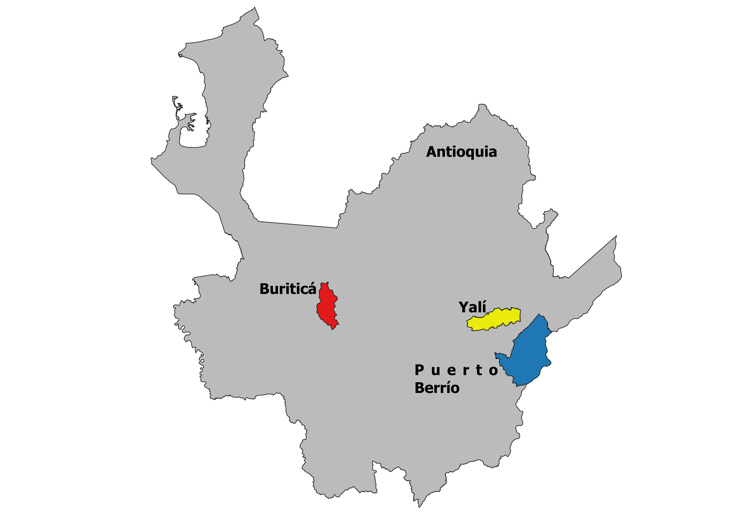


**Fig. S1** Study areas. Colombia (Green). Antioquia (Gray). Buriticá (Red). YalÍ (Yellow). Puerto Berrío (Blue).


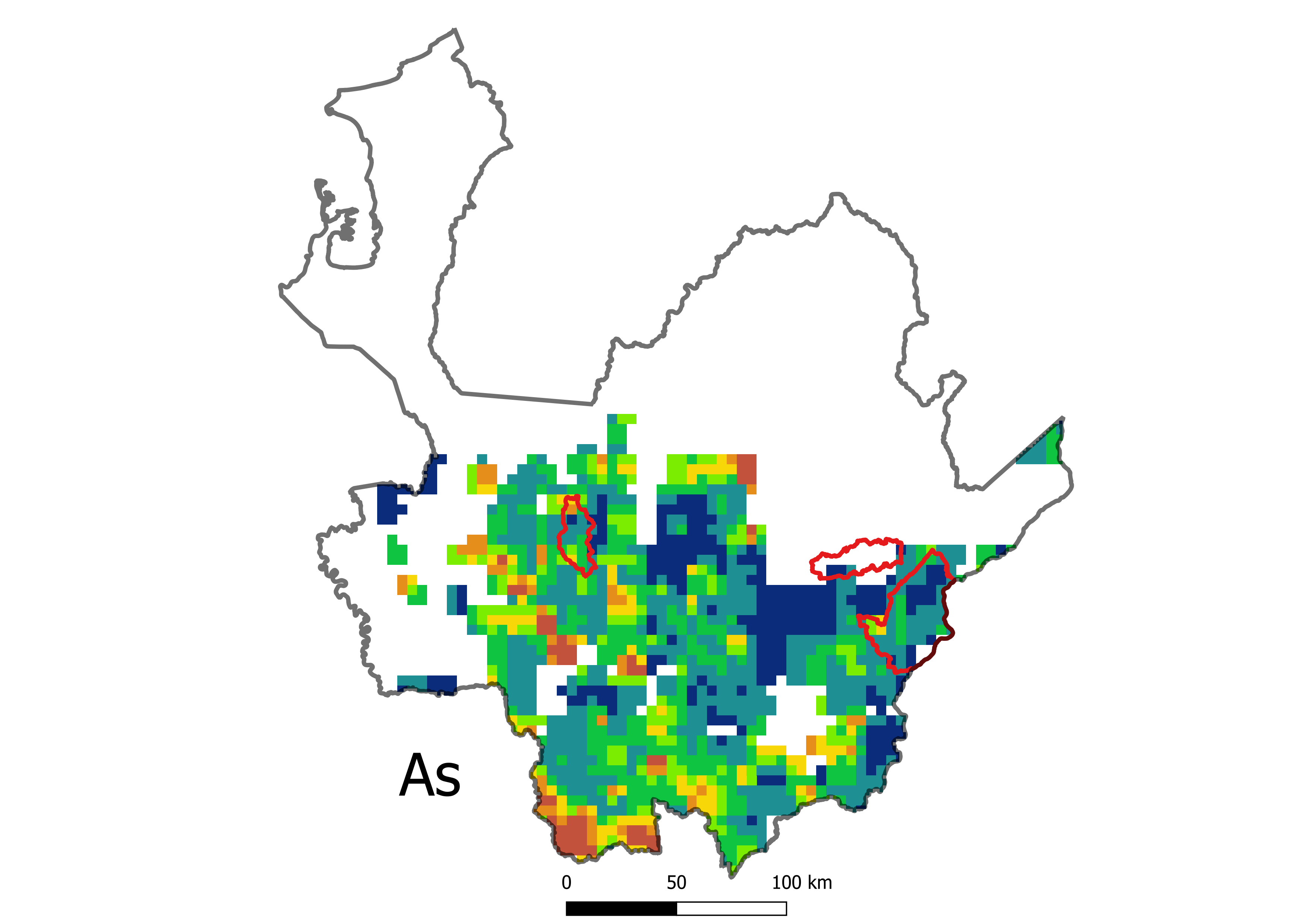

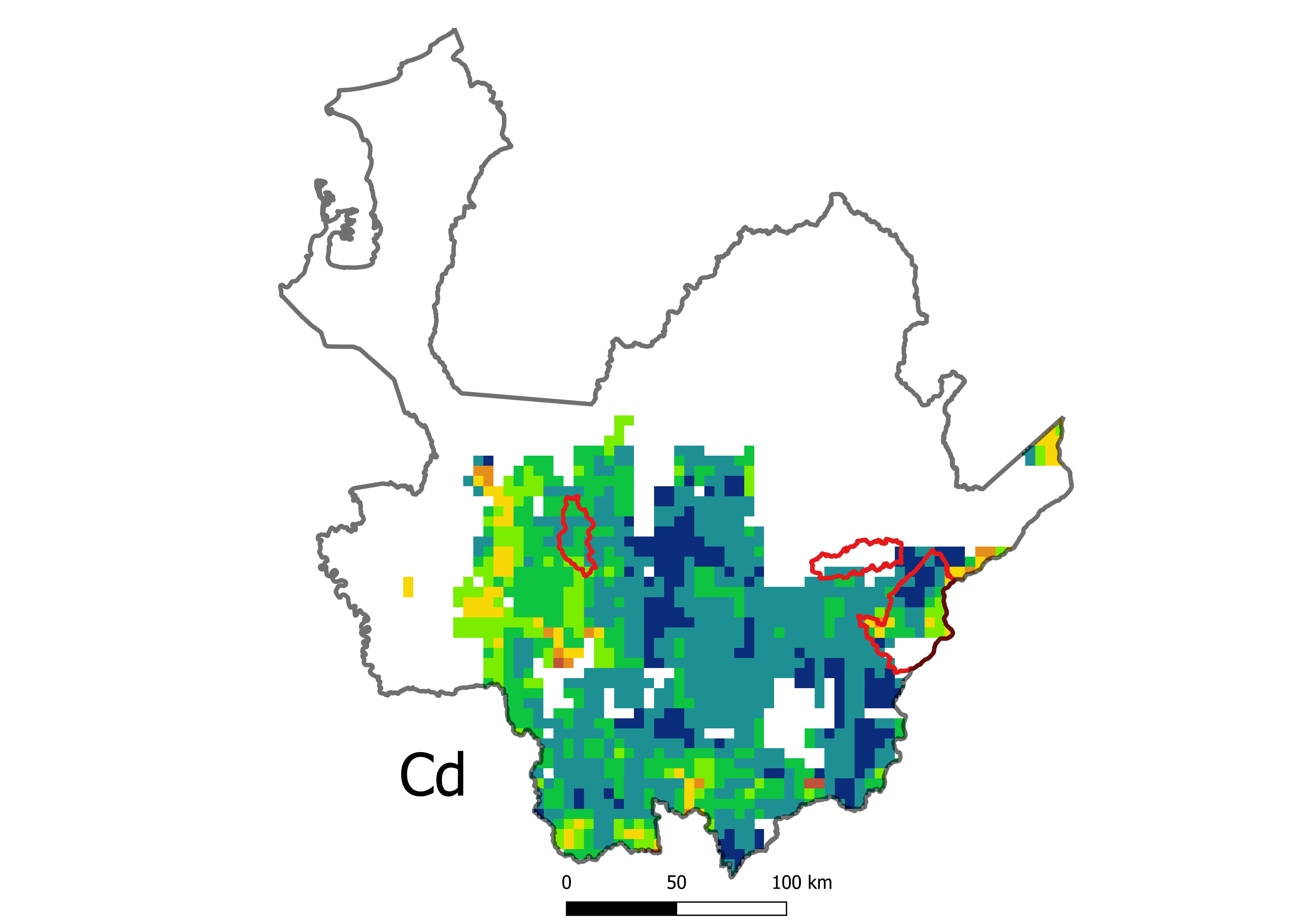

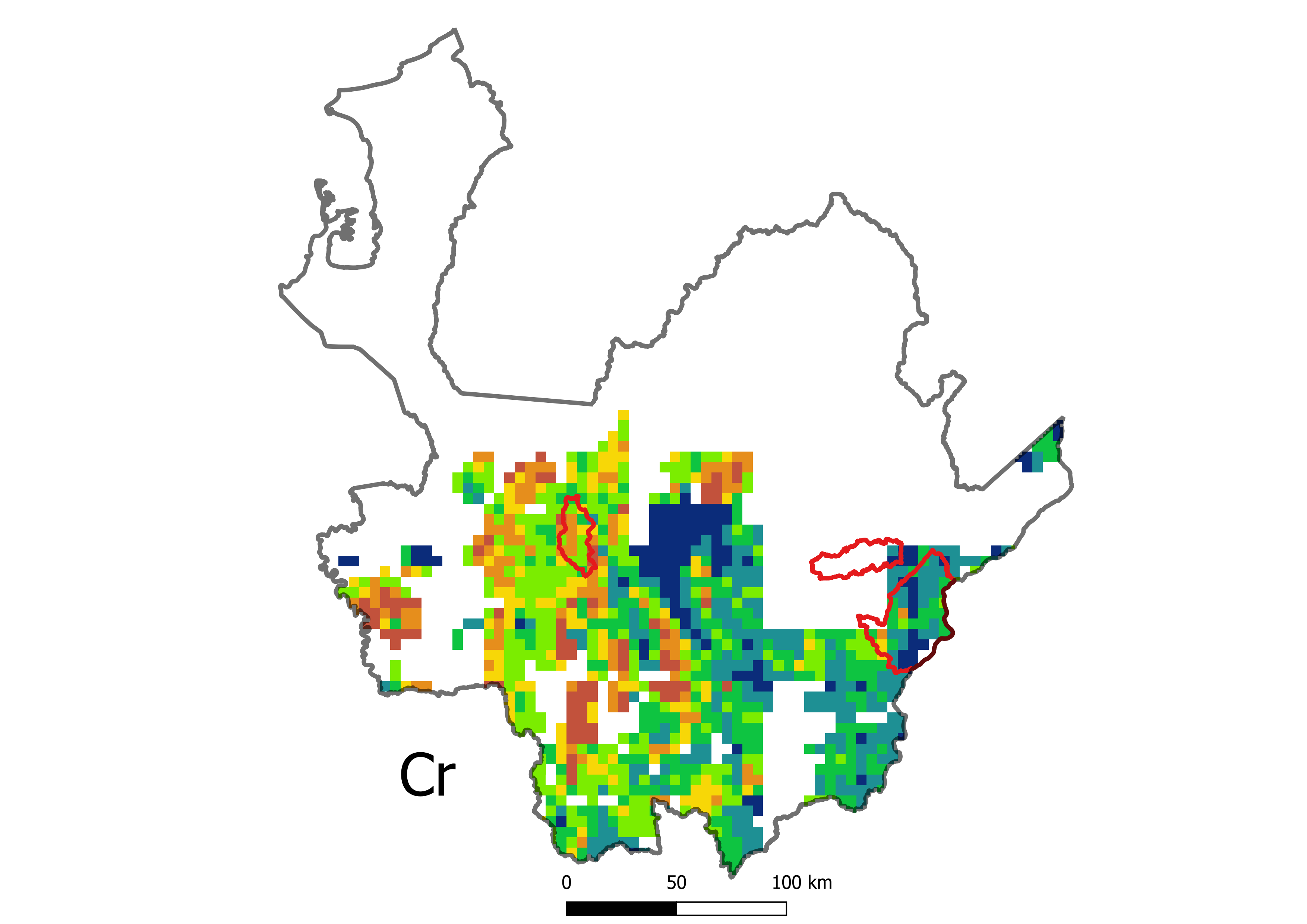

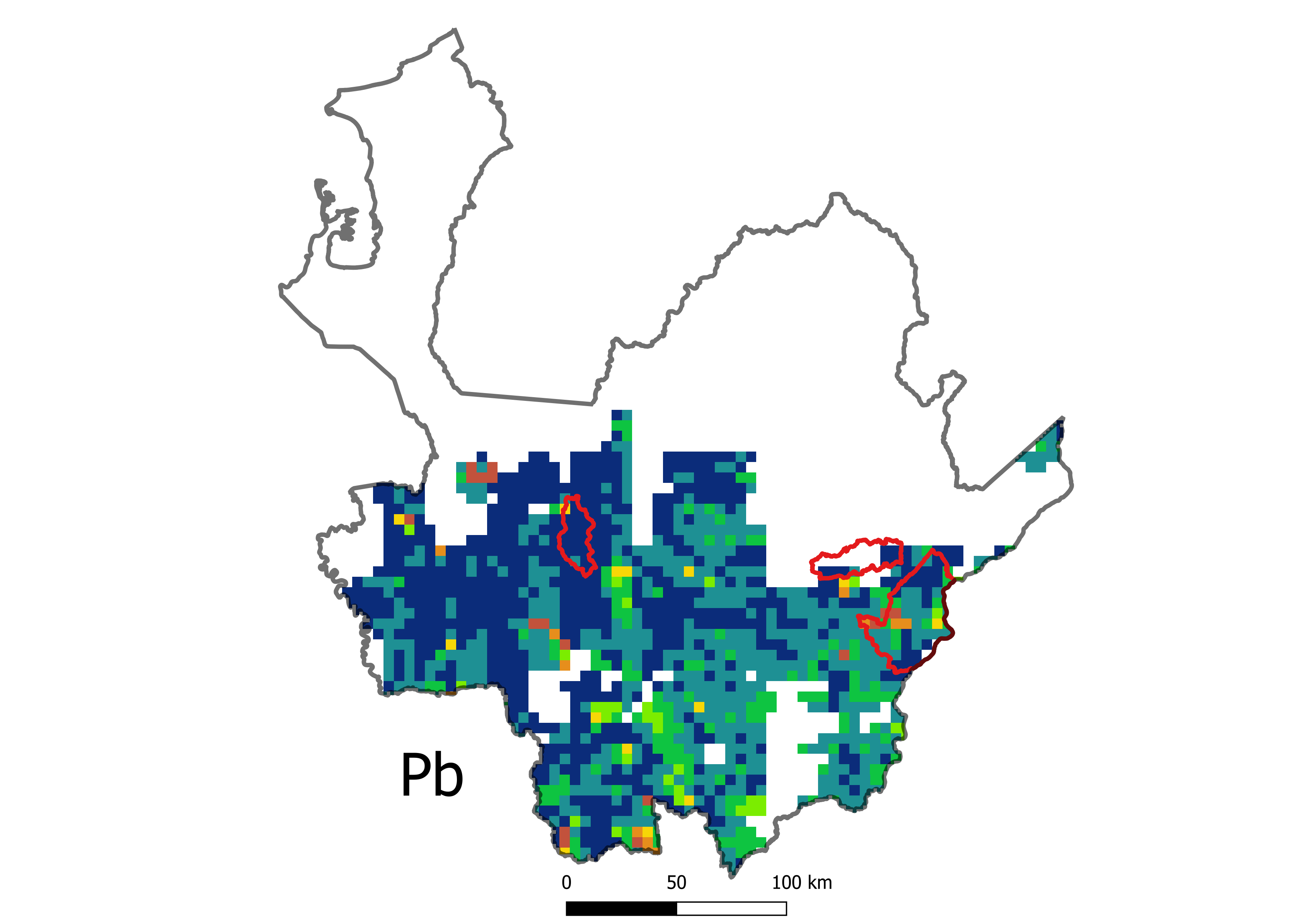


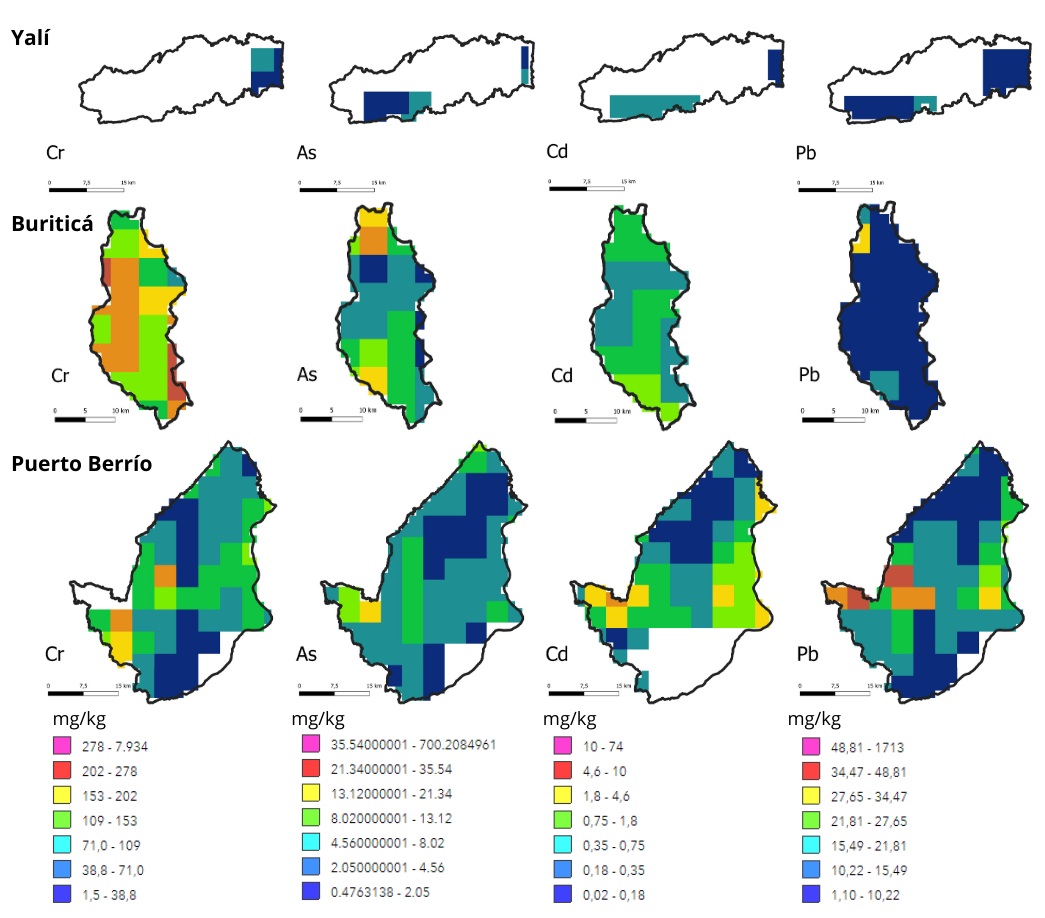


**Fig. S2** Geochemical maps for Yalí, Puerto Berríom and Buriticá. The data was obtained from the geochemical map of Colombia, which is available as [[https://srvags.sgc.gov.co/JSViewer/Atlas_geoquimico_2016/#](https://srvags.sgc.gov.co/JSViewer/Atlas_geoquimico_2016/)].

**
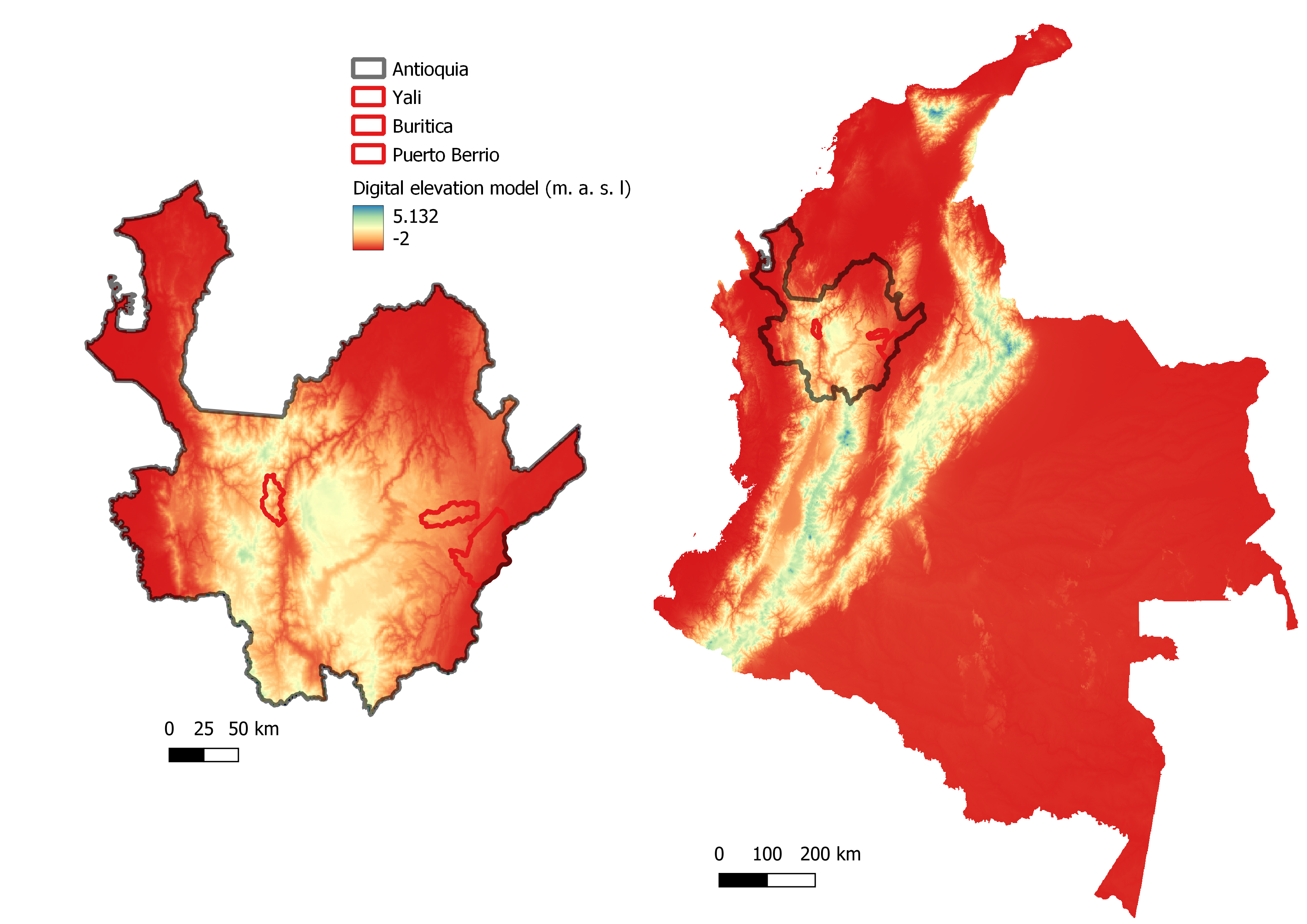
**

**Fig. S3** Digital elevation model for Colombia.

Supplementary description S1

Buriticá: municipality located on the eastern margin of the western mountain range. The gold mineralization is found within the so-called Buriticá Tonalite associated with the Sabanalarga Batholith of reported Mesozoic age. This unit forms N-S elongated bodies across the Cauca River. The structural control is N-S associated with the Cauca and Tonusco faults.

Puerto Berrio: located in the lower part of the eastern slope of the central mountain range of Colombia. Three lithological units can be distinguished: Neis feldspathic, the Segovia batholith and the East Segovia sedimentite of Mesozoic age. Structurally, the gold mining area is affected by the Otú, Palestina, Cimitarra and El Bagre faults, locally in the Minas del Vapor sector the faults have a predominant NW trend; the mineralization of vetiform orogenic gold type.

Yalí: located on the eastern slope of the central mountain range. The gold mineralization is associated with the geological unit known as Batolito Antioqueño with variable composition of Tonalite, Granodiorite and Diorite, all Mesozoic age. This geological body has structural controls to the east by the Palestina fault system and to the west by the Romeral fault system. Gold mineralization is in the form of veinlets.

Supplementary description S2

Four certified synthetic solutions for trace elements ((Millipore Sigma) were used for ICP-OES calibration. Calibration curve was accepted at R^2^ > 0.9996. The LOD (mg L^-1^) for Pb, As, Cr, and Cd were 0.089, 0.028, 0.041, and 0.007, respectively. The LOQ (mg L^-1^) for Pb, As, Cr, and Cd were 0.015, 0.09, 0.013, and 0.003, respectively. Certificated reference materials, NIST 2709a San Joaquin soil and BCR® 701 lake sediment, were included in each digestion/extraction batch for quality assurance. For soil samples the recoveries of metals calculated relative to the certified concentration ranged between 90 and 103%. Duplicate samples were included to evaluate reproducibility. The coefficients of variation for each set of duplicate reference samples ranges from 0.8 to 9.8% (average 5.3%). Certified synthetic solutions for ICP-OES and blank were included in each batch for quality assurance. All reagents were of analytical grade, glassware was soaked in acid (3% HNO_3_) bath overnight prior use.

**Table S1.** Background values in the soil.

|  | **As** | **Cd** | **Cr** | **Pb** |
| --- | --- | --- | --- | --- |
| Background level |  |  |  |  |
| Soils | 6.0 | 0.35 | 70 | 35 |
| Soil forming rocks | 2.0 | 0.15 | 20.0 | 18.0 |

(Sparks et al. 2013; Gallego and Olivero-Verbel 2021).

**Table S2**. Calculated values for Pollution Index (PI) associated with concentrations of As, Cd, Pb, and Cr for the three municipalities under study.

| Area | Sampling points | PI | | | |
| --- | --- | --- | --- | --- | --- |
|  |  | As | Cd | Pb | Cr |
| Yalí | 1 | 2,0 | 4,0 | 0,5 | 0,3 |
| Yalí | 2 | 1,7 | 2,9 | 0,6 | 0,3 |
| Yalí | 3 | 1,8 | 4,0 | 0,5 | 0,3 |
| Yalí | 4 | 1,7 | 2,9 | 0,6 | 0,3 |
| Yalí | 5 | 2,0 | 8,9 | 0,6 | 0,3 |
| Yalí | 6 | 4,6 | 13,2 | 3,1 | 1,6 |
| Yalí | 7 | 5,9 | 11,7 | 2,6 | 1,3 |
| Yalí | 8 | 5,4 | 12,3 | 2,9 | 1,4 |
| Yalí | 9 | 5,0 | 8,8 | 3,4 | 1,7 |
| Yalí | 10 | 2,0 | 3,1 | 0,6 | 0,3 |
| Yalí | 11 | 1,9 | 0,1 | 0,4 | 0,2 |
| Yalí | 12 | 1,8 | 5,7 | 0,6 | 0,3 |
| Yalí | 13 | 1,6 | 0,1 | 0,3 | 0,2 |
| Yalí | 14 | 2,2 | 5,7 | 0,5 | 0,3 |
| Yalí | 15 | 1,7 | 5,7 | 0,6 | 0,3 |
| Yalí | 16 | 1,4 | 0,1 | 0,3 | 0,1 |
| Yalí | 17 | 2,0 | 2,9 | 0,5 | 0,3 |
| Yalí | 18 | 2,2 | 3,7 | 0,5 | 0,2 |
| Yalí | 19 | 1,5 | 0,3 | 0,2 | 0,1 |
| Yalí | 20 | 1,7 | 0,5 | 0,2 | 0,1 |
| Yalí | 21 | 1,7 | 0,1 | 0,3 | 0,1 |
| Puerto Berrío | 1 | 148,7 | 186,3 | 8,0 | 4,0 |
| Puerto Berrío | 2 | 76,5 | 58,9 | 7,7 | 3,8 |
| Puerto Berrío | 3 | 50,3 | 125,1 | 7,0 | 3,5 |
| Puerto Berrío | 4 | 18,7 | 159,4 | 7,9 | 4,0 |
| Puerto Berrío | 5 | 83,0 | 67,7 | 8,6 | 4,3 |
| Puerto Berrío | 6 | 39,0 | 53,4 | 5,1 | 2,5 |
| Puerto Berrío | 7 | 25,8 | 68,3 | 6,0 | 3,0 |
| Puerto Berrío | 8 | 31,2 | 128,9 | 6,8 | 3,4 |
| Puerto Berrío | 9 | 6,0 | 48,9 | 3,7 | 1,9 |
| Puerto Berrío | 10 | 1,0 | 3,1 | 2,8 | 1,4 |
| Puerto Berrío | 11 | 16,4 | 6,6 | 8,5 | 4,3 |
| Puerto Berrío | 12 | 16,4 | 39,7 | 3,1 | 1,6 |
| Puerto Berrío | 13 | 1,0 | 27,7 | 2,2 | 1,1 |
| Puerto Berrío | 14 | 2,3 | 1,4 | 2,8 | 1,4 |
| Puerto Berrío | 15 | 0,3 | 0,3 | 2,0 | 1,0 |
| Puerto Berrío | 16 | 1,8 | 4,6 | 2,6 | 1,3 |
| Puerto Berrío | 17 | 7,7 | 1,7 | 1,9 | 0,9 |
| Puerto Berrío | 18 | 7,7 | 0,0 | 2,2 | 1,1 |
| Puerto Berrío | 19 | 17,6 | 14,9 | 2,9 | 1,5 |
| Puerto Berrío | 20 | 50,5 | 157,7 | 4,5 | 2,2 |
| Puerto Berrío | 21 | 3,1 | 0,1 | 2,9 | 1,4 |
| Puerto Berrío | 22 | 1,7 | 5,1 | 2,5 | 1,3 |
| Puerto Berrío | 23 | 0,6 | 0,6 | 2,0 | 1,0 |
| Buriticá | 1 | 5,8 | 40,6 | 2,1 | 1,0 |
| Buriticá | 2 | 4,0 | 15,4 | 0,6 | 0,3 |
| Buriticá | 3 | 8,3 | 29,7 | 2,5 | 1,3 |
| Buriticá | 4 | 6,6 | 45,4 | 2,0 | 1,0 |
| Buriticá | 5 | 4,8 | 9,1 | 1,0 | 0,5 |
| Buriticá | 6 | 7,4 | 52,0 | 2,3 | 1,2 |
| Buriticá | 7 | 1,8 | 1,4 | 0,3 | 0,1 |
| Buriticá | 8 | 3,1 | 12,9 | 1,2 | 0,6 |
| Buriticá | 9 | 5,5 | 27,4 | 2,3 | 1,1 |
| Buriticá | 10 | 3,8 | 17,4 | 1,4 | 0,7 |
| Buriticá | 11 | 1,3 | 9,7 | 0,2 | 0,1 |
| Buriticá | 12 | 6,8 | 23,4 | 2,9 | 1,5 |
| Buriticá | 13 | 3,6 | 12,6 | 0,6 | 0,3 |
| Buriticá | 14 | 3,3 | 11,1 | 0,9 | 0,5 |
| Buriticá | 15 | 1,0 | 7,1 | 0,4 | 0,2 |
| Buriticá | 16 | 1,8 | 15,7 | 0,6 | 0,3 |
| Buriticá | 17 | 5,6 | 42,6 | 2,2 | 1,1 |
| Buriticá | 18 | 1,1 | 10,0 | 0,4 | 0,2 |
| Buriticá | 19 | 3,8 | 9,1 | 1,0 | 0,5 |

**Table S3**. Results for biomonitoring.

| **Area** | **Sampling points** | **Germination percentage** (**%G)** | **Radicle Length (mm)** |
| --- | --- | --- | --- |
| Yalí | 1 | 60 | 1,4 ±0.3 |
| Yalí | 2 | 70 | 2,1 ±0.2 |
| Yalí | 3 | 70 | 1,8 ±0.0 |
| Yalí | 4 | 60 | 1,5 ±0,1 |
| Yalí | 5 | 70 | 2,1 ±0,3 |
| Yalí | 6 | 60 | 1,8 ±0,2 |
| Yalí | 7 | 60 | 1,5 ±0,0 |
| Yalí | 8 | 50 | 1,3 ±0,4 |
| Yalí | 9 | 60 | 1,9 ±0,2 |
| Yalí | 10 | 60 | 2,1 ±0,4 |
| Yalí | 11 | 80 | 3,5 ±0,6 |
| Yalí | 12 | 80 | 2,1 ±0,4 |
| Yalí | 13 | 80 | 2,8 ±0,0 |
| Yalí | 14 | 80 | 3,2 ±0,0 |
| Yalí | 15 | 70 | 2,5 ±0,1 |
| Yalí | 16 | 80 | 3,1 ±3,1 |
| Yalí | 17 | 70 | 2,7 ±0,1 |
| Yalí | 18 | 60 | 1,3 ±0,4 |
| Yalí | 19 | 70 | 1,6 ±0,2 |
| Yalí | 20 | 80 | 2,9 ±0,3 |
| Yalí | 21 | 80 | 3,1 ±0,3 |
| Puerto Berrío | 1 | 30 | 0,8 ±0,1 |
| Puerto Berrío | 2 | 50 | 1,6 ±0,1 |
| Puerto Berrío | 3 | 50 | 1,9 ±0,2 |
| Puerto Berrío | 4 | 30 | 1,2 ±0,5 |
| Puerto Berrío | 5 | 50 | 2,1 ±0,1 |
| Puerto Berrío | 6 | 60 | 2,4 ±0,1 |
| Puerto Berrío | 7 | 60 | 1,9 ±0,0 |
| Puerto Berrío | 8 | 30 | 1,3 ±0,1 |
| Puerto Berrío | 9 | 50 | 2,1 ±0,1 |
| Puerto Berrío | 10 | 70 | 2,5 ±0,2 |
| Puerto Berrío | 11 | 70 | 2,8 ±0,6 |
| Puerto Berrío | 12 | 70 | 2,3 ±0,3 |
| Puerto Berrío | 13 | 50 | 1,9 ± 0,1 |
| Puerto Berrío | 14 | 70 | 2,2 ±0,1 |
| Puerto Berrío | 15 | 70 | 2,4 ±0,2 |
| Puerto Berrío | 16 | 70 | 2,5 ±0,4 |
| Puerto Berrío | 17 | 70 | 2,0 ±0,1 |
| Puerto Berrío | 18 | 70 | 2,1 ±0,2 |
| Puerto Berrío | 19 | 50 | 1,6 ±0,0 |
| Puerto Berrío | 20 | 50 | 1,8 ±0,0 |
| Puerto Berrío | 21 | 70 | 2,9 ±0,1 |
| Puerto Berrío | 22 | 70 | 2,5 ±0,0 |
| Puerto Berrío | 23 | 70 | 2,8 ±0,2 |
| Buriticá | 1 | 50 | 1,1 ±0,5 |
| Buriticá | 2 | 60 | 2,4 ±0,4 |
| Buriticá | 3 | 40 | 2,5 ±0,5 |
| Buriticá | 4 | 50 | 1,2 ±0,2 |
| Buriticá | 5 | 60 | 2,8 ±0,5 |
| Buriticá | 6 | 40 | 2,3 ±0,7 |
| Buriticá | 7 | 60 | 2,3 ±0,6 |
| Buriticá | 8 | 60 | 2,4 ±0,1 |
| Buriticá | 9 | 50 | 1,3 ±0,0 |
| Buriticá | 10 | 70 | 2,5 ±0,0 |
| Buriticá | 11 | 60 | 2,4 ±0,2 |
| Buriticá | 12 | 60 | 2,1 ±0,0 |
| Buriticá | 13 | 60 | 2,3 ±0,7 |
| Buriticá | 14 | 60 | 2,5 ±0,2 |
| Buriticá | 15 | 80 | 2,6 ±0,2 |
| Buriticá | 16 | 70 | 2,2 ±0,4 |
| Buriticá | 17 | 40 | 2,1 ±0,0 |
| Buriticá | 18 | 70 | 2,6 ±0,2 |
| Buriticá | 19 | 70 | 2,3 ±0,1 |

**Table S4.** Spearman's rank correlation coefficient analysis between potential toxic elements concentration (As, Cd, Pb, and Cr), length, germination percentage (G%), plant vigor index (PVI), pollution index (PI) for each potential toxic elements, and pollution load index (PLI).

| Variable | for Variable | Spearman's ρ | Probab > \|ρ\| |
| --- | --- | --- | --- |
| Cd | As | 0,75 | <,0001 |
| Pb | As | 0,75 | <,0001 |
| Pb | Cd | 0,67 | <,0001 |
| Cr | As | 0,77 | <,0001 |
| Cr | Cd | 0,77 | <,0001 |
| Cr | Pb | 0,60 | <,0001 |
| G% | As | -0,65 | <,0001 |
| G% | Cd | -0,78 | <,0001 |
| G% | Pb | -0,55 | <,0001 |
| G% | Cr | -0,71 | <,0001 |
| Length | As | -0,45 | 0,0024 |
| Length | Cd | -0,56 | <,0001 |
| Length | Pb | -0,46 | 0,0014 |
| Length | Cr | -0,36 | 0,0154 |
| Length | G% | 0,79 | <,0001 |
| PVI | As | -0,55 | 0,0001 |
| PVI | Cd | -0,67 | <,0001 |
| PVI | Pb | -0,50 | 0,0005 |
| PVI | Cr | -0,49 | 0,0006 |
| PVI | G% | 0,90 | <,0001 |
| PVI | Length | 0,96 | <,0001 |
| PLI | As | 0,81 | <,0001 |
| PLI | Cd | 0,92 | <,0001 |
| PLI | Pb | 0,65 | <,0001 |
| PLI | Cr | 0,92 | <,0001 |
| PLI | G% | -0,75 | <,0001 |
| PLI | Length | -0,47 | 0,0011 |
| PLI | PVI | -0,59 | <,0001 |
| As PI | As | 0,99 | <,0001 |
| As PI | Cd | 0,72 | <,0001 |
| As PI | Pb | 0,74 | <,0001 |
| As PI | Cr | 0,75 | <,0001 |
| As PI | G% | -0,63 | <,0001 |
| As PI | Length | -0,45 | 0,0019 |
| As PI | PVI | -0,55 | <,0001 |
| As PI | PLI | 0,81 | <,0001 |
| Cd PI | As | 0,73 | <,0001 |
| Cd PI | Cd | 0,99 | <,0001 |
| Cd PI | Pb | 0,67 | <,0001 |
| Cd PI | Cr | 0,76 | <,0001 |
| Cd PI | G% | -0,77 | <,0001 |
| Cd PI | Length | -0,56 | <,0001 |
| Cd PI | PVI | -0,66 | <,0001 |
| Cd PI | PLI | 0,92 | <,0001 |
| Cd PI | As PI | 0,70 | <,0001 |
| Pb PI | As | 0,77 | <,0001 |
| Pb PI | Cd | 0,77 | <,0001 |
| Pb PI | Pb | 0,60 | <,0001 |
| Pb PI | Cr | 0,87 | <,0001 |
| Pb PI | G% | -0,71 | <,0001 |
| Pb PI | Length | -0,36 | 0,0154 |
| Pb PI | PVI | -0,49 | 0,0006 |
| Pb PI | PLI | 0,92 | <,0001 |
| Pb PI | As PI | 0,75 | <,0001 |
| Pb PI | Cd PI | 0,76 | <,0001 |
| Cr PI | As | 0,77 | <,0001 |
| Cr PI | Cd | 0,77 | <,0001 |
| Cr PI | Pb | 0,60 | <,0001 |
| Cr PI | Cr | 0,98 | <,0001 |
| Cr PI | G% | -0,71 | <,0001 |
| Cr PI | Length | -0,36 | 0,0154 |
| Cr PI | PVI | -0,49 | 0,0006 |
| Cr PI | PLI | 0,92 | <,0001 |
| Cr PI | As PI | 0,75 | <,0001 |
| Cr PI | Cd PI | 0,76 | <,0001 |
| Cr PI | Pb PI | 0,87 | <,0001 |

**Table S5.** Cubic Cluster Criterion (CCC). Cluster analysis based on the concentration of PTEs.

| Nº conglomerate | CCC |
| --- | --- |
| 1 | 0.00 |
| 2 | -0.11 |
| 3 | 3.02 |
| 4 | 4.50 |
| 5 | 5.88 |
| 6 | 9.49 |
| 7 | 11.52 |

**Table S6.** Cubic Cluster Criterion (CCC). Cluster analysis based on the concentration of PLI index.

| Nº conglomerate | CCC |
| --- | --- |
| 1 | 0.00 |
| 2 | 1.20 |
| 3 | 3.44 |
| 4 | 1.72 |
| 5 | 1.91 |
| 6 | 1.98 |
| 7 | 2.41 |

**Table S7.** Cubic Cluster Criterion (CCC). Cluster analysis based on the concentration of PVI index.

| Nº conglomerate | CCC |
| --- | --- |
| 1 | 0.00 |
| 2 | 2.76 |
| 3 | 3.21 |
| 4 | 3.78 |
| 5 | 3.19 |
| 6 | 3.27 |
| 7 | 3.70 |

**References**

Gallego JL, Olivero-Verbel J (2021) Cytogenetic toxicity from pesticide and trace element mixtures in soils used for conventional and organic crops of Allium cepa L. Environ Pollut 276:116558. https://doi.org/10.1016/j.envpol.2021.116558

Provoost J, Cornelis C, Swartjes F (2006) Comparison of soil clean-up standards for trace elements between countries: Why do they differ? J Soils Sediments 6:173–181. https://doi.org/10.1065/jss2006.07.169

Sparks DL, (2013) Environmental Soil Chemistry, 2nd ed, Journal of Chemical Information and Modeling. Academic Press, San Diego, California. <https://doi.org/10.1017/CBO9781107415324.004>
